# Chemical Structure and Biological Activities of Secondary Metabolites from *Salicornia europaea* L.

**DOI:** 10.3390/molecules26082252

**Published:** 2021-04-13

**Authors:** Sojeong Kim, Eun-Young Lee, Prima F. Hillman, Jaeyoung Ko, Inho Yang, Sang-Jip Nam

**Affiliations:** 1 Graduate School of Industrial Pharmaceutical Sciences, Ewha Womans University, Seoul 03760, Korea; ksj9558@gmail.com; 2 Department of Chemistry and Nanoscience, Ewha Womans University, Seoul 03760, Korea; younglee0124@naver.com (E.-Y.L.); primafitriah@gmail.com (P.F.H.); 3 AMOREPACIFIC Research and Development Center, Yongin 17074, Korea; jaeyoungko@amorepacific.com; 4 Department of Convergence Study on the Ocean Science and Technology, Korea Maritime and Ocean University, Busan 49112, Korea

**Keywords:** *Salicornia europaea* L., halophyte, phytochemicals, secondary metabolites

## Abstract

*Salicornia europaea* L. is a halophyte that grows in salt marshes and muddy seashores, which is widely used both as traditional medicine and as an edible vegetable. This salt-tolerant plant is a source of diverse secondary metabolites with several therapeutic properties, including antioxidant, antidiabetic, cytotoxic, anti-inflammatory, and anti-obesity effects. Therefore, this review summarizes the chemical structure and biological activities of secondary metabolites isolated from *Salicornia europaea* L.

## 1. Introduction

*Salicornia europaea* L., also known as *Salicornia herbacea* L., is a halophyte belonging to the Chenopodiaceae subfamily with many common names including glasswort, sea beans, sea asparagus, and samphire [1]. As implied by its names, this edible plant tolerates up to 3% salinity [2] and grows in salt marshes and muddy seashores in temperate and subtropical regions worldwide, including the western and southern coasts of Korea [3]. In Asia, *S. europaea* has been traditionally used as a traditional medicine for constipation, nephropathy, hepatitis, diarrhea, obesity, and diabetes, among other disorders [4,5]. Moreover, in addition to using *S. europaea* for glass making, its aerial parts are used in salads, pickles, fermented food, and salt substitutes [6,7]. Therefore, due to the many health benefits of *S. europaea*, several studies proposed the development of this halophyte as a functional food and medicinal plant. Importantly, *S. europaea* crude extracts reportedly possess several therapeutic properties. For instance, the presence of immunomodulatory compounds in crude extracts was demonstrated via RAW 264.7 macrophage cell line assays [8]. Moreover, the antioxidant activity of *S. europaea* was measured via the 2,2-diphenyl-1-picrylhydrazyl (DPPH) radical scavenging, hydroxyl radical scavenging, and reactive oxygen species (ROS) generation assays [9]. The antihyperglycemic and antihyperlipidemic activities of this plant were also demonstrated in mice fed with a high-fat diet [10] and another study reported that a polysaccharide extract from this plant exhibited anti-inflammatory activity in vitro and in vivo [11].

In addition to the many studies that have characterized the bioactivity of crude *S. europaea* extracts or individual fractions, many bioactive secondary metabolites have been isolated from this plant. Therefore, this review sought to systematically classify the secondary metabolites isolated from *S. europaea* according to their chemistry and summarize their biological activities. Most of the biological activities of the secondary metabolites discussed herein were taken from separate studies. Many studies generally classify glasswort-like species as *S. europaea* due to the difficulties of taxonomic identification of the genus *Salicornia.* Therefore, it is worth noting that this manuscript is by no means a comprehensive review of all *S. europaea* studies, and instead focuses on representative examples.

## 2. Oleanane Triterpenoid Saponins

**Figure 1:**
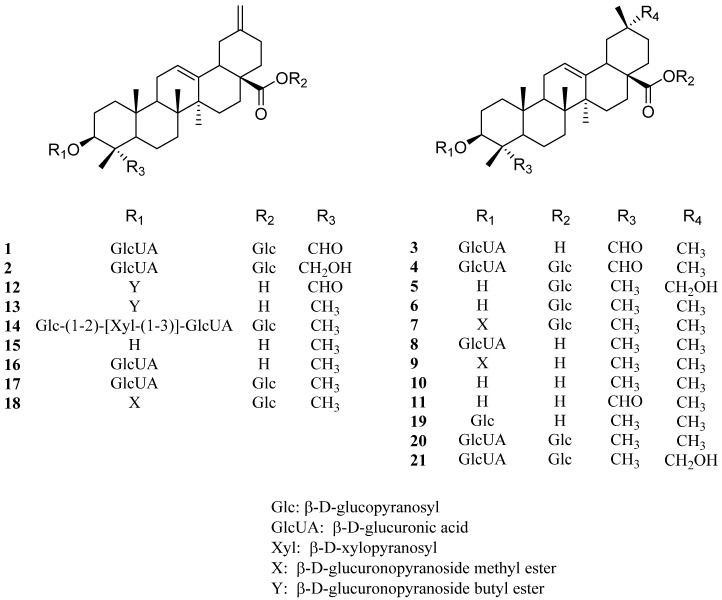
Chemical structures of oleanane triterpenoid saponins isolated from *Salicornia europaea* (**1**–**21**).

Triterpenoid saponins are abundant in plants and possess a variety of biological activities, including antifungal, antiviral, antioxidant, antiglycation, and anticancer properties [12,13,14,15]. More than twenty oleanane-type triterpenoid saponins, including 30-noroleanane triterpenoid saponins, have so far been isolated from *S. europaea* (Figure 1).

In 2012, a new 30-noroleanane triterpenoid saponin, 3*β*-hydroxy-23-oxo-30-noroleana-12,20(29)-diene-28-oic acid 3-*O*-*β*-d-glucuronopyranosyl-28-*O*-*β*-d-glucopyranoside (**1**), and three known triterpenoid saponins (**2**–**4**) were also isolated from an *n*-BuOH fraction of an *S. europaea* extract [5]. The known compounds were identified as 30-norhederagenin 3-*O*-*β*-d-glucuronopyranosyl-28-*O*-*β*-d-glucopyranoside (**2**), gypsogenin 3-*O*-*β*-d-glucuronopyranoside (**3**), and gypsogenin 3-*O*-*β*-d-glucuronopyranosyl-28-*O*-*β*-d-glucopyranoside (**4**). The antioxidant activities of compounds **1**–**4** have been evaluated by measuring their 1,1-diphenyl-2-picryl-hydrazyl (DPPH) radical and peroxynitrite (ONOO^−^) scavenging activities. All four compounds were found to be potent scavengers for both authentic peroxynitrite and peroxynitrite produced by morpholinosydnonimine (SIN-1) (IC_50_ between <<1 and 21.9 μM). Notably, compound **2** had the lowest IC_50_ values (<<1 μM) for both authentic ONOO^−^ and ONOO^−^ produced by SIN-1, but displayed no significant DPPH radical scavenging activity. Compound **1** also possesses a potent antifungal activity against *Colletotrichum gloeosporioides* [16]. Compound **1** has been previously isolated from *Salicornia bigelovii*. Compound **3** has been isolated from the flowering plant *Gypsophila pacifica* and exhibited promising hepatoprotective activity [17]. This triterpenoid saponin has also been isolated from fruits of *Acanthopanax senticosus* and showed pancreatic lipase inhibitory activity (Table 1) [18].

In the same year, Yin et al. reported the isolation and structural elucidation of 3*β*,29-dihydroxyolean-12-en-28-oic acid 28-*O*-*β*-d-glucopyranosyl ester (**5**), a new oleanane triterpenoid saponin, along with four previously identified compounds: oleanolic acid 28-*O*-*β*-d-glucopyranoside (**6**), chikusetsusaponin IVa methyl ester (**7**), calenduloside E (**8**), and calenduloside E 6ʹ-methyl ester (**9**) [19]. The plant sample in question was collected in Jiangsu Province, China, along the Yellow Sea shore. Although the biological activity of **5** has not yet been described, the bioactivities of compounds **6**–**9** have been studied extensively. Compound **6**, isolated from *Drypetes paxii* reportedly exhibits antibacterial activity [20], and an isolate from the plant *Aralia cordata* has been reported to possess anti-inflammatory effects [21]. A synthesized version of compound **6** also displayed potent α-glucosidase and α-amylase inhibitory activities [22]. The reported biological effects of compound **7** include anticancer [23,24,25], antifungal [16], and anti-inflammatory [26] activities. Compound **8** exhibited potent spermicidal [27] and anticancer activities [23,28]. Compounds **7** and **8** have been isolated from *Gardenia jasminoides* roots [23], whereas compound **9** has been isolated from *Salicornia bigelovii*, *Acanthopanax sessiliflorus*, and *Ilex rotunda*, and were reported to possess cytotoxic activity [29,30] and a moderate anticlotting effect [31].

In 2014, Zhao et al. identified two known oleanane-type terpenoid saponins, oleanolic acid (**10**) and gypsogenin (**11**), along with two new 30-noroleanane triterpenoid saponins, salbige A (**12**) and B (**13**), in a *S. europaea* methanol extract [32]. Interestingly, the *S. europaea* analyzed in this study was collected from the salt lake of Xinjiang Province in China, which is thousands of kilometers from the nearest ocean. Compounds **12** and **13** displayed potent anti-proliferative activities against A549 cancer cells in the aforementioned study, with IC_50_ values of 52.35 and 79.39 μM, respectively. The biological activities of compound **10**, which is one of the most abundant and well-known triterpenoid saponins, included antioxidant, antitumor, anti-inflammatory, antidiabetic, antimicrobial, hepatoprotective, antihypertensive, antiparasitic [33], and antiviral [34] activities. This versatile pentacyclic triterpenoid is abundant and conspicuous in plants of the Oleaceae family, including the olive plant, and have been identified in a wide range of plants including *Achyranthes aspera*, *Aspilia africana*, *Lantana camara*, *Ocimum sanctum*, *Vitis vinifera*, *Flaveria trinervia*, *Syzygium aromaticum*, and *Miconia albicans* [33]. Synthetically prepared compound **11** exhibited antimicrobial, antiproliferative, and apoptotic effects [35].

In 2018, Lyu et al. reported the isolation of five more 30-noroleanane triterpenoid saponins from whole *S. europaea* plants, including the previously undescribed compound salieuropaea A (3-*O*-*β*-glucopyranosyl-(1→2)-[*β*-xylopyranosyl-(l→3)]-*β*-glucuronopyranosyl 30-noroleanolic acid 28-*O*-*β*-glucopyranosyl ester, **14**) [36]. The other isolated 30-noroleanane triterpenoid saponins were identified as akebonic acid (**15**), boussingoside A_1_ (**16**), boussingoside A_2_ (**17**), and 3-*O*-[*β*-d-glucuronopyranosyl-6′-*O*-methyl ester]-30-norolean-12,20(29)-dien-28-*O*-[*β*-d-glucopyranosyl] ester (**18**). Nine oleanane triterpenoid saponins (**5**–**10** and **19**–**21**), including oleanolic acid 3-*O*-*β*-d-glucopyranoside (**19**), chikusetsusaponin IVa (**20**), and zygophyloside K (**21**), were also isolated and structurally characterized in this study. The plant sample was collected from Jiangsu Province, China. Although the biological activities of compounds **14** and **17** have not been described yet, compound **15** was reported to exhibit anti-HIV-1 protease activity [37], antibacterial activity [38], an inhibitory effect on Aβ42-induced fibrillogenesis [39], an α-glucosidase inhibitory effect, and moderate in vitro cytotoxic activity against human cancer cell lines [40]. Compound **15** has been isolated mostly from the flowering plant family Lardizabalaceae, such as *Stauntonia obovatifoliola*, *Akebia quinata*, and *Akebia trifoliata* [37,38,39,40]. Compound **16**, which was previously isolated from the Colombian climbing plant *Boussingaultia baselloides*, was found to exhibit hypoglycemic activity in rats [41]. Compound **18** has displayed cytotoxic activity towards the SK-N-SH and HL60 cell lines [42], as well as a potent inhibitory activity against *Colletotrichum gloeosporioides* [16]. Thiyagarajan et al. reported that vitalboside A (**19**) isolated from *Syzygium cumini* could be a potent therapeutic agent to manage obesity and diabetes due to its inhibitory effect on PTP1B and partial agonism of the peroxisome proliferator-activated receptor γ [43]. Compound **20** has been reported to possess antiviral [44], antithrombotic [45], insulinotropic [46], anti-inflammatory [47], and anti-obesity [48] activities. The presence of compound **20** has been reported in other plants, including *Alternanthera philoxeroides* [44], *Ilex paraguariensis* [45], and *Dolichos lablab* seeds [48]. No studies have been reported regarding the bioactivity of compound **21**.

## 3. Caffeoylquinic Acid Derivatives

**Figure 2:**
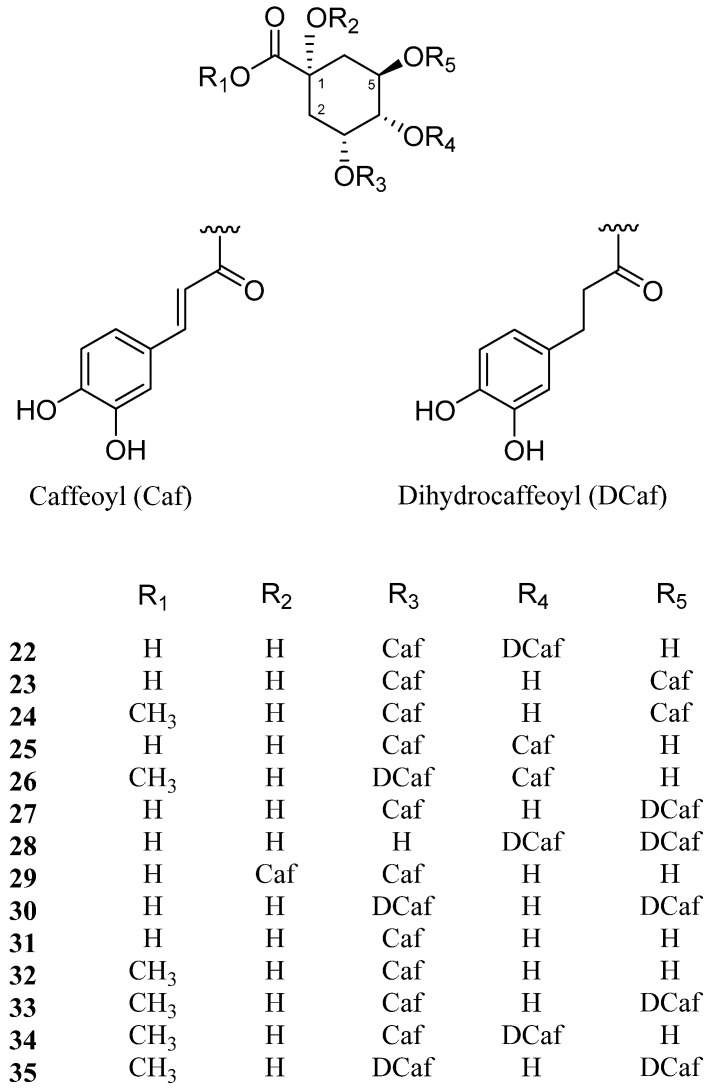
Chemical structures of caffeoylquinic acids isolated from *Salicornia europaea* (**22**–**35**).

Caffeoylquinic acid (CQA) derivatives have been reported in many plants including coffee beans, and their various biological activities include antioxidant, antibacterial, anticancer, and antihistaminic effects [49] (Figure 2).

In 2005, Chung et al. isolated and determined the structure of a new natural chlorogenic acid derivative, tungtungmadic acid (3-caffeoyl-4-dihydrocaffeoyl quinic acid, **22**) [50]. The plant materials were collected from Busan, in the southern coast of Korea. In this study, tungtungmadic acid displayed a strong antioxidant activity in both DPPH free radical scavenging and iron-induced liver microsomal lipid peroxidation inhibitory assays, with IC_50_ values of 5.1 and 9.3 μM, respectively (Table 2). Studies have also reported that compound **22** can protect plasmid DNA from hydroxyl radical-induced strand breakage. Several other studies on the biological activities of compound **22** have been conducted since its isolation. For instance, compound **22** has also been reported to provide protection against carbon tetrachloride (CC1_4_)-induced hepatic fibrosis and *tert*-butyl hydroperoxide (*t*-BHP)-induced hepatotoxicity [50,51]. Moreover, this compound possesses anti-inflammatory properties [52], inhibits tumor cell invasion [53], and prevents high-glucose-induced lipid accumulation in human HepG2 cells [54]. Interestingly, the occurrence of compound **22** has not been reported in other sources (Table 2).

In 2011, Kim et al. reported the isolation of compound **22** and four other caffeoylquinic acid derivatives from *S. europaea* collected from Younggwang, southwestern coast of Korea [6]. The four known compounds were identified as 3,5-dicaffeoylquinic acid (**23**), methyl 3,5-dicaffeoylquinate (**24**), 3,4-dicaffeoylquinic acid (**25**), and the novel compound methyl 4-caffeoyl-3-dihydrocaffeoylquinate (salicornate, **26**). Importantly, all of these dicaffeoylquinic acid derivatives (**22**–**26**) were found to possess significant antioxidant activities, as demonstrated by measurements of both DPPH radical scavenging and cholesteryl ester hydroperoxide (CE-OOH) formation inhibiting activities. Dicaffeoylquinic acids (**23**, **25**) derived from *Youngia japonica*, a biannual medicinal herb, also exhibited antibacterial activities [55]. Similarly, a dicaffeoylquinic acid methyl ester form (**24**) isolated from the aerial parts of *Ageratina adenophora* exhibited antibacterial activity against *Salmonella enterica* [56]. Moreover, extracts from the edible plant *Centella asiatica* exhibited neuroprotective activity in in vitro models of Aβ toxicity, which includes compounds **23** and **25** [57]. An independent study in 2012 also demonstrated the neuroprotective activity of compounds **23**–**25**, which were isolated from *Ilex latifolia* [58]. Furthermore, compounds **23**–**25** could be used to treat diabetes and diabetic complications, and were identified in other plant sources including *Artemisia capillaris*, *Gynura divaricata*, and *Artemisia iwayomogi* [59,60,61]. Compound **23** was also found in *Laggera alata*, *Artemisia capillaris*, *Helichrysum populifolium*, and *Erycibe obtusifolia* and was reported to possess antithrombotic activity [62], anti-inflammatory effects [63], hepatoprotective and antiviral activity [64,65,66,67], and cytotoxic activity [68]. Similarly, compound **24** exhibits a range of bioactivities, including anti-inflammatory [63,69,70], antitumor [71,72], and anti-melanogenic [73] effects. Compound **25** has been found to possess antithrombotic [62], antihyperlipidemic [74], and antiviral [65,66,67] activities. Interestingly, compound **25** was also found in the fruits of *Pandanus tectorius*, a mangrove plant [74].

In 2015, two new (**27**, **28**) and four known caffeoylated quinic acids (**23**, **24**, **29**, **30**) were isolated from *S. europaea*, and their potential to alleviate high mobility group box 1 (HMGB1)-mediated vascular barrier disruption was evaluated in vitro and in vivo [75]. In this study, farm-raised plant material was obtained from Shinan, which is located on the southern coast of Korea. The new compounds were identified as 3-*O*-caffeoyl-5-*O*-dihydrocaffeoyl quinic acid (**27**) and 4,5-di-*O*-dihydrocaffeoyl quinic acid (**28**), along with the known compounds 1,3-di-*O*-caffeoyl quinic acid (**29**) and 3,5-di-*O*-dihydrocaffeoyl quinic acid (**30**). According to this study, compounds **23**, **27**, **29**, and **30** showed vascular protective activities against inflammatory responses induced by HMGB1 in both cellular and animal models. Compound **29** has also been reported to possess antioxidant and cytoprotective effects [76]. This study also showed that the positions of two caffeoyl groups were closely related to their activity. Among the di-*O*-caffeoylquinic acid compounds, entities with adjacent caffeoyl moieties exhibited better performance than their non-adjacent configured counterparts.

In 2016, Cho et al. reported the isolation of three known (**27**, **31**, **32**) and three new caffeoylquinic acid derivatives (**33**–**35**) from methanol extracts of *S. europaea* collected from Younggwang, in the southwestern coast of Korea [77]. The known compounds included 3-caffeoylquinic acid (**31**) and 3-caffeoylquinic acid methyl ester (**32**), and the new compounds were established as 3-caffeoyl-5-dihydrocaffeoylquinic acid methyl ester (**33**), 3-caffeoyl-4-dihydrocaffeoylquinic acid methyl ester (**34**), and 3,5-di-dihydrocaffeoylquinic acid methyl ester (**35**). In this study, all six compounds scavenged DPPH radicals and inhibited CE-OOH formation. The dicaffeoylquinic acid derivatives (**27**, **33**–**35**), which have two catechol groups, showed higher activities than the mono-caffeoylquinic acid derivatives (**31**, **32**) and caffeic acid did. Additionally, compound **31** extracted from leaves of *Moringa oleifera* reportedly possesses moderate influenza A neuraminidase inhibitory activity [78]. This compound also exerts neuroprotective properties via the inhibition of pro-inflammatory responses in activated microglia [79].

## 4. Flavonoids and Flavanones

**Figure 3:**
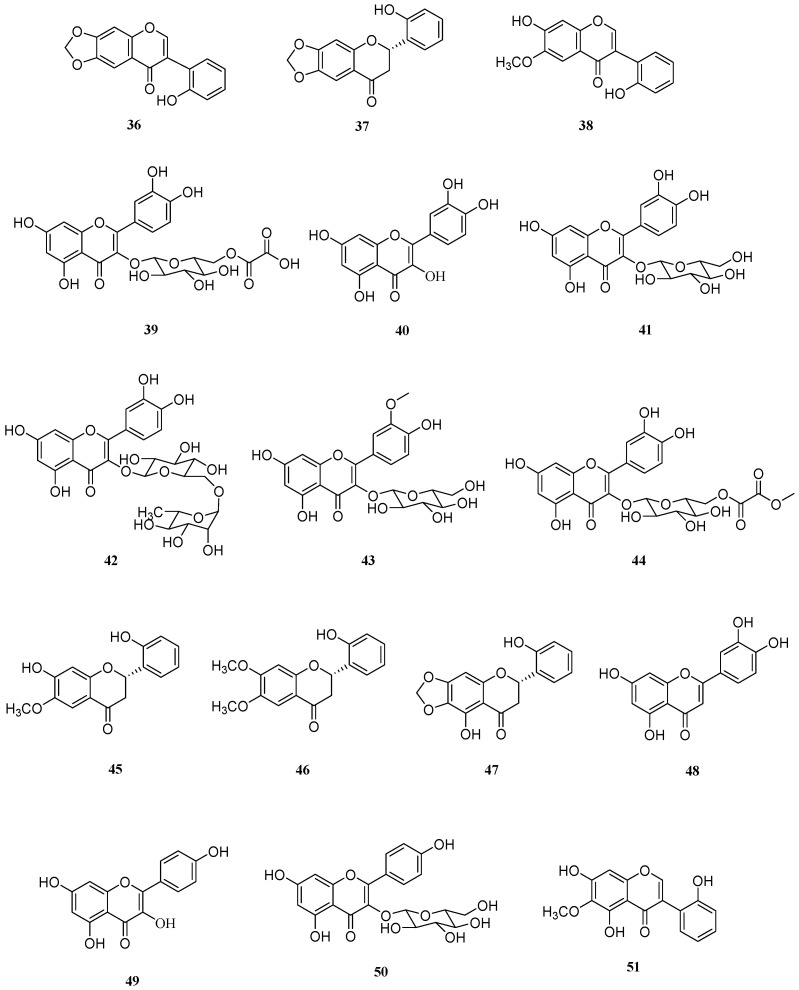
Chemical structures of flavonoids isolated from *Salicornia europaea* (**36**–**51**).

Flavonoids and flavonoid glycosides have also been isolated from *S. europaea* (Figure 3). In 1982, Arakawa et al. reported the isolation and structural elucidation of 2′-hydroxy-6,7-methylenedioxyisoflavone (**36**), (−)-(2*S*)-2′-hydroxy-6,7-methylenedioxyflavanone (**37**), and 2′,7-dihydroxy-6-methoxyisoflavone (**38**) from a methanol extract of *S. europaea* [80]. The plants for this study were collected from lake Notoro, which is a coastal lagoon by the northern shore of Hokkaido, Japan.

Three years later, Geslin et al. isolated quercetin 3-*O*-(6″-*O*-malonyl)-*β*-d-glucoside (**39**), quercetin (**40**), quercetin 3-*O*-*β*-d-glucopyranoside (**41**), rutin (**42**), and isorhamnetin 3-*O*-*β*-d-glucopyranoside (**43**) from *S. europaea* collected from Loire-Atlantique, by the Bay of Biscay, France [81]. A cherry blossom derived compound (**39**) reportedly acted as a potent suppressor of the production of advanced glycation end products (AGEs) and fibroblast apoptosis by AGEs [82]. This quercetin isolated from the leaves of *Corchorus olitorius* or the fruit peel of *Sicana odorifera* acted as an antioxidative agent [83,84]. A broad range of biological effects has been reported for compounds **40**–**42**, including anti-inflammatory, antidiabetic, cardiovascular protection, and anticancer effects [85,86,87]. Compound **43**, isolated from *S. europaea*, has been reported to be a potential agent for the prevention and/or treatment of diabetes [88] and a chemo preventive agent for cancer [89], as well as an anti-oxidant [90] and anti-obesity agent (Table 3) [91].

Kim and Park isolated compounds **41** and **43** in 2004 from plant samples collected from Muan-gun, southwestern coast of Korea, and demonstrated that the antioxidant activities of compounds **41** and **42** were similar to those of compound **40** [92]. However, the activity of compound **43**, which contains a methoxy group on the flavonoid B ring, was lower than the activity of compound **40**.

In 2011, Kim et al. reported the isolation and antioxidant activities of a novel flavonoid glycoside, isoquercitrin 6″-*O*-methyloxalate (**44**), along with the known compounds **41** and **43** [6]. Compounds **41** and **44**, which have no substitutions on their B rings, exhibited significant antioxidant activities, whereas the antioxidant activity of compound **43** was comparatively less potent. These results agreed with previous reports that the catechol group of the B ring plays an important role in determining the antioxidant activities of flavonoids [93,94].

In 2015, two new flavanones and one known flavanone (**45**–**47**) were isolated by Tuan et al. from an ethyl acetate extract of farm-raised *S. europaea* [95]. The isolated compounds were identified as 2*S*-2′,7-dihydroxy-6-methoxyflavanone (**45**), 2*S*-2′-hydroxy-6,7-dimethoxy-flavanone (**46**), and 2*S*-5,2′-dihydroxy-6,7-methylenedioxyflavanone (**47**). The authors assessed the suppressive activities of compounds **45**–**47** against HMGB1 and found that they inhibited both LPS-stimulated HMGB1 secretion in vitro and cecal ligation and puncture (CLP)-induced HMGB1 secretion in vivo. Moreover, compound **47**, which was previously isolated from *Iris* spp., also exhibited promising antiglycation activity [96].

Three flavonoids (**48**–**50**) that are widely known for their pharmacological activities have been isolated from *S. europaea*, in addition to compounds **40**–**43** [36]. Luteolin (**48**) has been found to display antioxidant, antitumor, anti-inflammatory, antiapoptotic, and cardioprotective activities [97]. This well-known flavonoid is one of the most intensely studied plant-derived metabolites, which is abundant in carrots, cabbage, tea, and apples. Kaempferol (**49**) is a hydroxyl group regioisomer of compound **48**. Compound **50** is a glucoside derivative of **49**. The biological activities of kaempferol (**49**) and kaempferol-3-*O*-*β*-d-glucoside (**50**) include anti-inflammatory, antioxidant, neuroprotective, cardioprotective, antidiabetic, and anticancer properties [98,99]. Compound **50**, also known as astragalin, is found in a wide range of medicinal plants such as wild garlic, tea, Chinese bittersweet, and sundew [99].

Irilin B (**51**) was recently identified and isolated from *S. europaea* via antioxidant activity-guided isolation and purification [100]. Compound **51** exhibits a good antioxidant and anti-neuroinflammatory potential. Moreover, compound **51** derived from *Chenopodium procerum*, an African medicinal plant, reportedly displayed antifungal activity against the plant pathogenic fungus *Cladosporium cucumerinum* [101]. Interestingly, another study reported the estrogenic activity of this compound from *Iris songarica* [102].

## 5. Chromones

**Figure 4:**
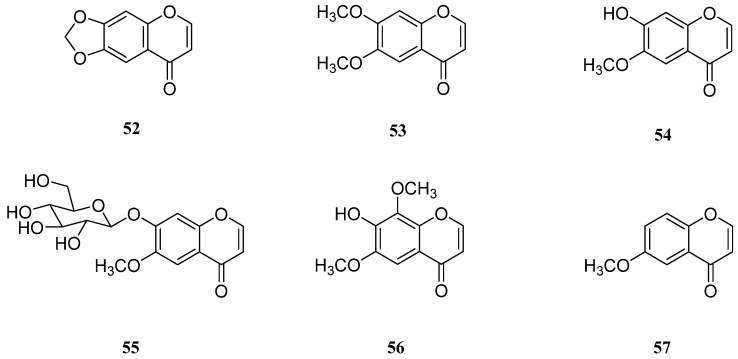
Chemical structures of chromones isolated from *Salicornia europaea* (**52**–**57**).

Chromones are benzoannelated γ-pyrone heterocycles that are widely found in nature, particularly in plants (Figure 4). Several pharmacological properties of chromones, including their anti-allergic, anti-inflammatory, antidiabetic, antitumor, and antimicrobial effects, have been identified thus far [103,104].

In 1978, two new naturally occurring 2,3-unsubstituted chromones, 6,7-methylenedioxychromone (**52**) and 6,7-dimethoxychromone (**53**), were isolated from the leaves and stems of *S. europaea* collected from the Notoro lakeside in Japan [105].

Five years later, Arakawa et al. identified and characterized the chromones 7-hydroxy-6-methoxychromone (**54**) and 7-*O*-*β*-d-glucopyranosyl-6-methoxychromone (**55**) from a methanol extract of *S. europaea*, neither of which had been previously identified in natural sources [106].

Most recently, Tuan et al. isolated a new naturally occurring chromone, 7-hydroxy-6,8-dimethoxychromone (**56**) along with compound **53** and 6-methoxychromanone (**57**) from farm-raised *S. europaea* [95]. In this study, compounds **53**, **56**, and **57** inhibited the release of HMGB1, which resulted in improved survival rates of CLP murine models. However, bioactivity study of these chromes are relatively unexplored, as shown in Table 4.

## 6. Sterols

**Figure 5:**
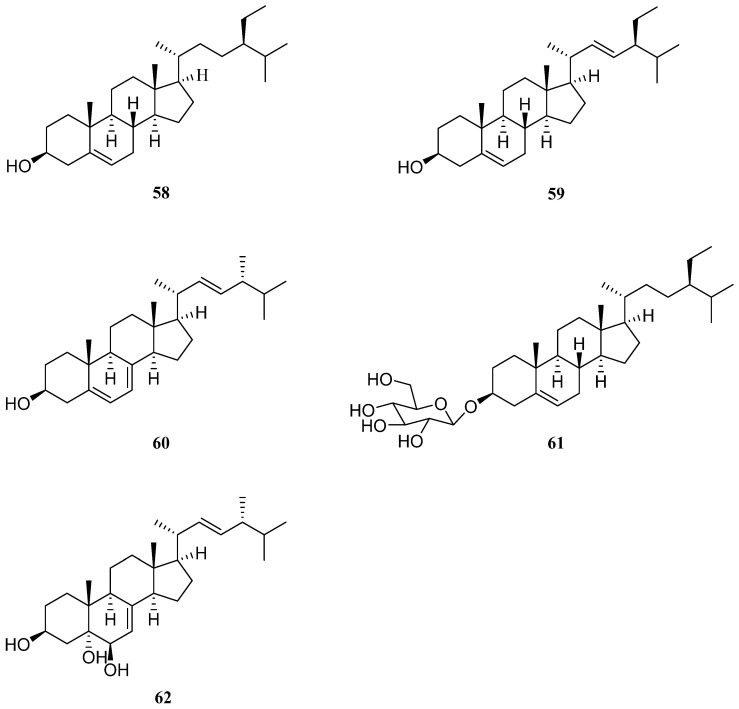
Chemical structures of sterols isolated from *Salicornia europaea* (**58**–**62**).

Sterols are part of the vast isoprenoid family of compounds and are essential for all eukaryotes [107]. Five sterols have been isolated from *S. europaea*, including β-sitosterol (**58**), stigmasterol (**59**), ergosterol (**60**), β-daucosterol (**61**), and cerevisterol (**62**) (Figure 5).

In 2004, Lee et al. isolated and identified compounds **58** and **59** [108]. In this study, plant samples were collected from Mokpo, on the southwestern coast of Korea. Compound **58** has been shown to possess anti-inflammatory, anticancer, hypocholesterolemic, immunomodulatory, antioxidant, neuroprotective, and antidiabetic effects (Table 5) [109]. Moreover, this compound is among the predominant phytosterols in the human diet, along with campesterol and stigmasterol. Furthermore, compound **59** displays anti-osteoarthritic, anti-hypercholesterolemic, cytotoxic, antitumor, hypoglycemic, antioxidant, antimutagenic, and anti-inflammatory properties (Table 5) [110]. This compound was first isolated from *Physostigma venenosum*, a poisonous native tropical plant, but has thereafter been identified in other medicinal plants such as *Croton sublyratus*, *Ficus hirta*, *Eclipta alba*, *Eclipta prostrate*, and *Parkia speciosa*. Wang et al. isolated compounds **59** and **60** from *S. europaea* collected from Jiangsu Province, China [111], whereas Lyu et al. isolated compounds **58**, **59**, **61**, and **62** from the same plant species collected from a different location in Jiangsu Province [36]. Compounds **60** and **62** derived from the edible mushroom *Cantharellus cibarius* have been found to possess potent NF-κB inhibitory activities (Table 5) [112]. However, compound **62** isolated from *Agaricus blazei*, also known as almond mushroom, has been reported to exert cytotoxic effects [113]. This compound was also isolated from another mushroom genus *Trametes* and was found to exhibit antimicrobial effects, as well as antibiotic resistance modifying activity [114]. Compound **61** exhibits immunoregulatory [115], anti-inflammatory [116], and anticancer properties [117], and reportedly promotes the proliferation of neural stem cells (Table 5) [118].

## 7. Lignans

**Figure 6:**
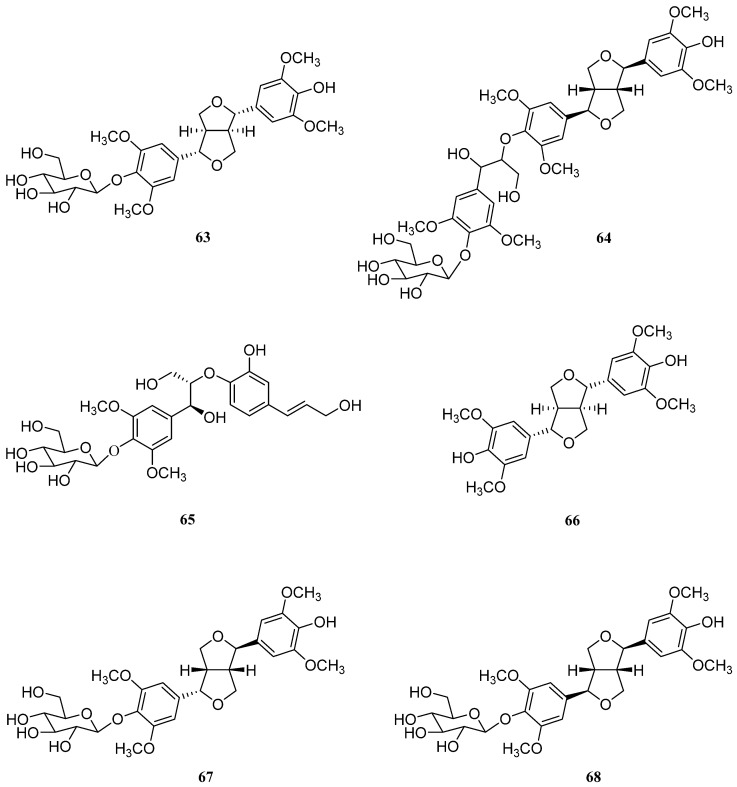
Chemical structures of sterols isolated from *Salicornia europaea* (**63**–**68**).

Lignans are broadly distributed in the plant kingdom and possess significant pharmacological properties, including anti-inflammatory, antitumor, immunosuppressive, cardioprotective, antioxidant, and antiviral activities [119] (Figure 6).

In 2011, Wang et al. reported the isolation and identification of syringaresinol 4-*O*-*β*-d-glucopyranoside (**63**), erythro-1-(4-*O*-*β*-d-glucopyranosyl-3,5-dimethoxyphenyl)-2-syringaresinoxyl-propane-1,3-diol (**64**), and longifloroside B (**65**) from *S. europaea* [120]. Although the bioactivities of compounds **64** and **65** have not been described yet, compound **63** has been shown to possess several potent biological activities, including DPPH radical scavenging activity (Table 6) [121], antiestrogenic activity against MCF-7 cells [122], and antitumor activity against the A549 cancer cell line [123]. Compound **63** was found in the stem bark of *Albizzia julibrissin* [121], the Thai medicinal plant *Capparis flavicans* [122], and leaves from the *Fatsia japonica* plant [123]. This compound also has been found to modulate lipid and glucose metabolism in HepG2 cells and C2C12 myotubes (Table 6) [124].

In addition to compounds **64** and **65**, Lyu et al. isolated lignans, (−)-syringaresinol (**66**) and episyringaresinol-4′′-*O*-*β*-d-glucopyranoside (**67**) from *S. europaea* [36]. Compound **66** has been found to possess antiplatelet aggregation [125], DPPH radical scavenging, nitric oxide (NO) inhibition [126], and P-glycoprotein inhibition [127] activities. Recently, the cosmeceutical potential of the compound was demonstrated with a series of *in-vitro* experiments showing antiphotoaging properties [128]. This compound was also discovered in Formosan *Zanthoxylum simulans* [125], *Wikstroemia indica* roots [126], and *Sasa borealis* whole plant extracts [127], respectively. Moreover, tortoside A (**67**) extracted from the Asian medicinal plant *Millettia pulchra* exhibited a moderate NQO1-inducing effect (Table 6) [129].

Recently, Karthivashan et al. identified and isolated acanthoside B (**68**) from *S. europaea* and reported that is possessed antioxidative, anticholinergic, anti-neuroinflammatory, and anti-amnesic properties [130].

## 8. Aliphatic Compounds

**Figure 7:**
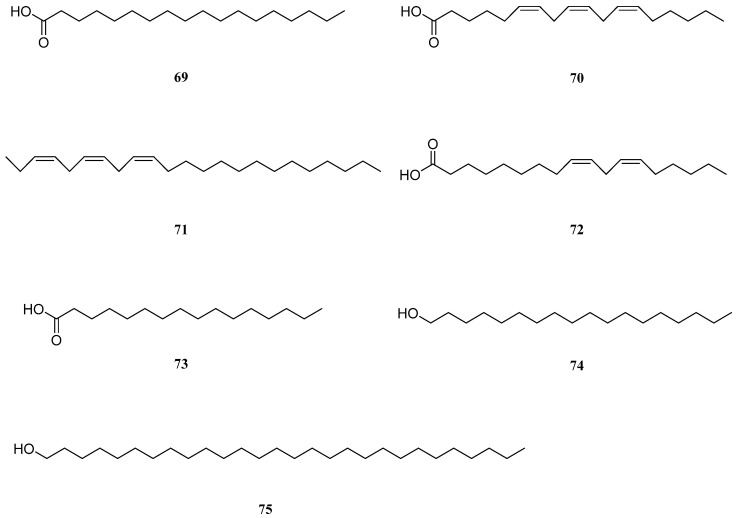
Chemical structures of aliphatic compounds isolated from *Salicornia europaea* (**69**–**75**).

Seven aliphatic compounds have been isolated from *S. europaea*: stearic acid (**69**), γ-linolenic acid (**70**), (3*Z*,6*Z*,9*Z*)-tricosa-3,6,9-triene (**71**), linoleic acid (**72**), hexadecanoic acid (**73**), 1-octadecanol (**74**), and 1-octacosanol (**75**) [36,111] (Figure 7). Compounds **69** and **73** are saturated fatty acids, compounds **70** and **72** are omega-6 polyunsaturated fatty acids, compound **71** is a polyunsaturated linear hydrocarbon, and compounds **74** and **75** are aliphatic alcohols. Wang et al. isolated compounds **69**–**72** and investigated their antioxidant and antiproliferative activities towards HepG2 and A549 cells. Interestingly, none of these compounds displayed a strong antioxidant activity except for compound **72**, which exerted a potent antiproliferative effect against both HepG2 and A549 cells (EC_50_ values of 65.35 ± 1.22 μM and 83.23 ± 3.26 μM, respectively). Compound **72**, an essential omega-6 fatty acid, also exhibits anti-inflammatory activity and has been used to treat rheumatoid arthritis, eczema, premenstrual syndrome, and diabetic neuropathy (Table 7) [131].

Compounds **73**–**75** have been isolated from *S. europaea* [36]. Compound **75** exhibits several pharmacological activities, including lipid-lowering, antiaggregatory, cytoprotective [132], and antiparkinsonian effects [133]. Compound **75**, 1-octacosanol, has also been reported to alleviate stress and restore stress-affected sleep in mice [134].

## 9. Others

**Figure 8:**
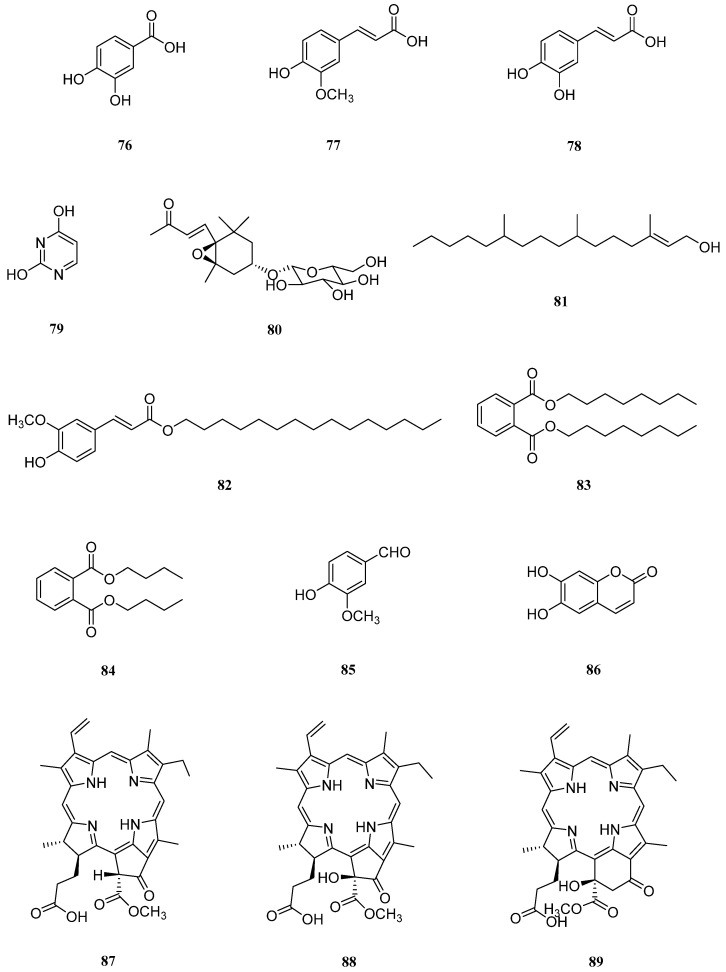
Chemical structures of isolates from *Salicornia europaea* (**76**–**89**).

In addition to the oleanane triterpenoid saponins, caffeoylquinic acid derivatives, flavonoids, chromones, sterols, lignans, and aliphatic compounds mentioned above, several other compounds have been isolated from *S. europaea* (Figure 8).

In 2007, Oh et al. conducted antioxidant assay-guided isolation and identified three phenolic compounds, protocatechuic acid (**76**), ferulic acid (**77**), and caffeic (**78**) acid, in *S. europaea* harvested from an abandoned salt farm in Haenam, southwestern coast of Korea (Table 8) [135]. Compounds **76**–**78** displayed significant DPPH, superoxide, and hydroxyl radical scavenging activities in this study. In addition to their antioxidant activities, compounds **76**–**78** exhibited a vast spectrum of other potent bioactivities. The reported pharmacological activities of compound **76** include antibacterial, antidiabetic, anticancer, anti-ulcer, antiaging, antifibrotic, antiviral, and anti-inflammatory effects [136]. Catechol benzoic acid (protocatechuic acid, **76**) is commonly found in grains and vegetables such as bran, brown rice, plums, and onion [136]. Compound **77** displays cholesterol-lowering, antimicrobial, anti-inflammatory, and anticancer activities, along with inhibitory effects against thrombosis and atherosclerosis (Table 8) [137]. This phenolic acid is abundant in plants and is usually found as an ester-linked form with polysaccharides from spinach, sugar beet, and bamboo. Compound **78** has been found to possess antibacterial, antiviral, anti-inflammatory, anti-atherosclerotic, immunostimulatory, antidiabetic, cardioprotective, antiproliferative, hepatoprotective, anticancer, antihepatocarcinoma, and antioxidant activities [138]. Caffeic acid (**78**) is a precursor of caffeine and is produced by a wide range of plants including olives, coffee beans, fruits, and potatoes (Table 8) [138].

Uracil (**79**) and icariside B2 (**80**) have also been isolated from *S. europaea* [108,120]. Compound **80** extracted from the Chinese desert-dwelling annual plant *Corispermum mongolicum* reportedly exhibits anti-inflammatory activity [139].

Moreover, in addition to phytol (**81**), dioctyl phthalate (**83**), dibutyl phthalate (**84**), vanillic aldehyde (**85**), and scopoletin (**86**), Wang et al. isolated a new compound, pentadecyl ferulate (**82**), from *S. europaea* collected from Jiangsu Province in China and elucidated its structure [111]. The antioxidant and antiproliferative activities of the isolated compounds were then investigated in this study. Compound **82** showed strong DPPH and superoxide radical scavenging activities (IC_50_ values of 27.6 ± 1.89 μM and 38.6 ± 2.23 μM, respectively) and inhibited the growth of both HepG2 and A549 cancer cells (EC_50_ values of 56 ± 2.32 μM and 48 ± 1.89 μM, respectively). Moreover, compound **81** exhibited selective antiproliferative activity against HepG2 cells (EC_50_ value of 78 ± 3.45 μM).

Compound **81** has also been reported to exert other biological functions, such as antimicrobial, cytotoxic, antioxidant, apoptosis- and autophagy-modulating, anxiolytic, anticonvulsant, immunomodulatory, antinociceptive, and anti-inflammatory activities [140]. However, phytol (**81**) is found in most plants as part of the chlorophyll molecule. Compound **83** has been shown to possess antibacterial [141], melanogenesis-inhibitory [142], and antioxidant activities, and reportedly exerts cytotoxic effects against the EACC cancer cell line [143]. This phthalate was isolated from a marine alga *Sargassum wightii*, *Nigella glandulifera* seeds, and the water hyacinth *Eichhornia crassipes*. Compound **84** exhibits antimicrobial [144,145,146], α-glucosidase inhibition [147], and cathepsin B inhibition activities [148]. Interestingly, dibutyl phthalate (**84**) was isolated from plants and bacterial sources such as *Ipomoea carnea*, *Begonia malabarica*, and *Streptomyces albidoflavus*, as well as *Streptomyces melanosporofaciens* and *Pseudomonas* sp. [144,145,146,147,148]. However, the debates about the origin of the phthalate should be considered whether the phthalates are natural products or accumulated contaminants.

Compound **85**, also known as vanillin, displays potent antimicrobial [149,150], antioxidant [151], and antidepressant activities [152]. A variety of pharmacological effects have been observed for scopoletin (**86**), which features a coumarin scaffold, as well as hepatoprotective [153], PC3 cell proliferation inhibitory [154], antioxidant [155], acetylcholinesterase inhibitory [156], hypouricemic [157], antifungal synergistic [158], immunomodulatory [159], antithyroid [160], anti-P-388 murine leukemia cell [161], hypoglycemic, hypolipidemic [162], and antiaging activities [163].

The pheophorbide compounds **87**–**89**, which are derivatives of chlorophyll a, have been isolated from a methanol extract of *S. europaea* [32]. Pheophorbide A (**87**) exhibits a strong antiproliferative activity against A549 and HepG2 cancer cell lines with IC_50_ values of 6.15 and 17.56 μM, respectively. (13^2^S)-Hydro-pheophorbide-lactone A (**89**) has been shown to possess a weak antioxidant activity, with a ferric reducing/antioxidant power (FRAP) value of 79.58 ± 1.69 mM/100 g and a DPPH scavenging rate of 75.33 ± 1.61%.

Compound **87** also possesses antitumor [164,165,166], photodynamic [167,168,169], and anti-inflammatory activities [170]. (13^2^S)-Hydroxy-pheophorbide A (**88**) exhibits potent photocytotoxicity, but its cytotoxic activity was reportedly lower than that of compound **87** [164,165]. However, compound **88** has a better anti-plasmodial performance than that of compound **87** [171]. The presence of a hydroxyl group at C-13 in compound **88** is the only structural difference between compounds **87** and **88.** Therefore, the hydroxyl substitution at the C-13 position might be responsible for the differences in the activities of these compounds.

In addition to the above-described compounds, *S. europaea* seeds have been reported to possess a wide range of fatty acids including compounds **69–73 [172]**.

## 10. Conclusions

*Salicornia europaea* is a popular salt-tolerant plant that has been traditionally used both as a functional food and vegetable seasoning; however, this plant is also known to produce compounds with therapeutic potential. Therefore, this plant is among the most widely recognized halophytes and is farmed in some regions to meet consumer demand.

This review discussed the chemistry and biological activities of *S. europaea* secondary metabolites reported from 1978 to October 2019. To the best of our knowledge, eighty-nine metabolites have been isolated, including oleanane triterpenoid saponins, caffeoylquinic acid derivatives, flavonoids, chromones, sterols, lignans, and aliphatic compounds. The diverse biological/pharmacological activities of the isolated compounds were also described in this review. Most of the compounds were obtained in small quantities ranges from 0.1 to 10 ppm (Table 9). Only a handful of the isolates were obtained over hundreds of ppm, including **1**, **39**, and **41** (123, 700, and 467 ppm, respectively). However, attention to the direct comparison of the yields is required as these high yields have resulted from dried plant material extraction.

Most of the plant samples discussed herein were collected from East Asia, including Korea, Japan, and China, where this plant has been historically used as food and for its therapeutic properties. Nonetheless, research on this plant is not strictly limited to Asia, but includes some regions of Europe as well. Moreover, the study of secondary metabolites from the genus *Salicornia* encompasses other temperate and subtropical regions worldwide, including America and Africa.

Previous studies on *S. europaea* have mainly focused on the identification of its secondary metabolites but often fail to provide other details. Specifically, most studies provide only basic descriptions of the collection sites and dates. However, the unique characteristics of this plant, including its seasonal color change, jointed segments, and scale-like stout leaves allow for its easy identification, which facilitates the isolation and identification of the enormous repertoire of secondary metabolites from this plant compared to that of other halophytes.

This review provides important insights that may facilitate the future study of the chemical profiles of this plant. For example, tungtungmadic acid (**22**) was exclusively isolated from the plant *S. europaea* and would be a good marker to identify this plant. Moreover, the chemical profile patterns of other secondary metabolites would provide useful references for chemists to identify and study this plant species. Therefore, we expect that future biochemical analyses of *S. europaea* and other halophytes will lead to the discovery of novel bioactive natural products.

## Figures and Tables

**Table 1:** Reported biological activities of compounds **1**–**21**.

No.	Biological Activities ^1^														
	Antioxidant			Antidiabetic	Cytotoxic							Antibacterial/ Antifungal		Anti-Inflammatory	Others
	**DPPH IC_50_ (μM)**	**Authentic ONOO¯ IC_50_ (μM)**	**SIN-1 IC_50_ (μM)**	**α-Glucosidase inhibitory IC_50_ (μM)**	**A2780 IC_50_ (μM)**	**HEY** **IC_50_ (μM)**	**HeLa IC_50_ (μM)**	**MCF-7 IC_50_ (μM)**	**A549 IC_50_ (μM)**	**A354-S2 IC_50_ (μM)**	**HepG2 IC_50_ (μM)**	**Antibacterial**	**Antifungal**		
**1**	>>5 × 10^2^	4.9	6.6										O		
**2**	3.9 × 10^2^	<<1	<<1												
**3**	>>5 × 10^2^	21.9	20.4												Pancreatic lipase inhibitory, hepatoprotective
**4**	>>5 × 10^2^	7.1	1.4												
**5**															
**6**				251.7	47.4	28.2						O		O	α-Amylase inhibitory
**7**					7.4	7.9	17.7	44.1	47.2	33.2			O	O	
**8**							5.4	56.0	97.5	29.9					Spermicidal
**9**							20.7	13.3	9.6	25.1					Anticlotting
**10**	O			O	O							O		O	Hepatoprotective, antihypertensive, antiparasitic, antiviral
**11**							22.5	9.0				O			
**12**									52.4						
**13**									79.4						
**14**															
**15**				9			27.8		48.8		51.9	O			Anti-HIV-1 protease, fibrillogenesis inhibitory
**16**				O											
**17**															
**18**					O								O		
**19**				O											PTP1B inhibitory
**20**				O										O	Antiviral, antithrombotic, insulinotropic, anti-obesity
**21**															

^1^ Qualitative bioassay study reported without quantitative data was marked with O.

**Table 2:** Reported biological activities of compounds **22**–**35**.

No.	Biological Activities ^1^						
	Antioxidant	Antidiabetic	Cytotoxic	Antibacterial	Anti-Inflammatory	Anti-HMGB1	Others
	**DPPH Scavenging IC_50_ (μM)**						
**22**	5.1		O		O		Hepatoprotective, lipogenesis inhibitory
**23**	6.1	O		O	O	O	Neuroprotective, antithrombotic, hepatoprotective, antiviral,
**24**	O	O	O	O	O	X	Neuroprotective, anti-melanogenic
**25**	3.4	O	O	O			Neuroprotective, antithrombotic, antihyperlipidemic, antiviral
**26**	O						
**27**	O					O	
**28**						X	
**29**	9.2		O			O	
**30**						O	
**31**	O						Influenza A neuraminidase inhibitory, neuroprotective
**32**	O						
**33**	O						
**34**	O						
**35**	O						

^1^ Qualitative bioassay study reported without quantitative data was marked with O.

**Table 3:** Reported biological activities of compounds **36**–**51**.

No.	Biological Activities ^1^					
	Antioxidant	Antidiabetic	Cytotoxic	Anti-Inflammatory	Anti-HMGB1	Others
		**AGE Production Inhibitory IC_50_ (μM)**				
**36**						
**37**						
**38**						
**39**	O	65.4				
**40**	O	105.9	O	O		Cardiovascular protection
**41**	O	64.6	O	O		Cardiovascular protection
**42**	O	O	O	O		Cardiovascular protection
**43**	O	O	O			anti-obesity
**44**	O					
**45**					O	
**46**					O	
**47**					O	Antiglycation
**48**	O		O	O		Antiapoptotic, cardioprotective
**49**	O	73.4	O	O		Neuroprotective, cardioprotective
**50**	O	227.5	O	O		Neuroprotective, cardioprotective
**51**	O			O		Antifungal, estrogenic

^1^ Qualitative bioassay study reported without quantitative data was marked with O.

**Table 4:** Reported biological activities of compounds **52**–**57**.

No.	Biological Activity ^1^
	**Anti-HMGB1**
**52**	
**53**	O
**54**	
**55**	
**56**	O
**57**	O

^1^ Qualitative bioassay study reported without quantitative data was marked with O.

**Table 5:** Reported biological activities of compounds **58**–**62**.

No.	Biological Activities ^1^					
	Antioxidant	Antidiabetic	Cytotoxic	Anti-Inflammatory	Anticancer	Others
		**AGE Production Inhibitory**				
**58**	O	O		O	O	Hypocholesterolemic, immunodulatory, neuroprotective
**60**	O		O	O		Anti-osteoarthritic, anti-hypercholesterolemic, antitumor, hypolgycemic, antimuatiogenic
**61**				O	O	Immunoregulatory, proliferation of neural stem cell
**62**						Antimicrobial, antibiotic

^1^ Qualitative bioassay study reported without quantitative data was marked with O.

**Table 6:** Reported biological activities of compounds **63**–**68**.

No.	Biological Activities ^1^		
	Antioxidant	Anti-Inflammatory	Others
	**DPPH Scavenging IC_50_ (μM)**		
**63**	10.5	O	Antiestrogenic, antitumor
**64**		O	
**65**		O	
**66**	19.5	O	Antiplatelet, nitric oxide inhibition, P-glycoprotein inhibition
**67**		O	NQO1-inducing
**68**	O	O	Anticholinergic, anti-neuroinflammatory, anti-amnesic

^1^ Qualitative bioassay study reported without quantitative data was marked with O.

**Table 7:** Reported biological activities of compounds **69**–**74**.

No.	Biological Activities ^1^			
	Antioxidant	Cytotoxic		Others
		**HepG2 EC_50_ (μM)**	**A549 EC_50_ (μM)**	
**69**	O	O		Antiproliferative
**70**	O	O		Antiproliferative
**71**	O	O		Antiproliferative
**72**	O	65.4	83.2	Antiproliferative
**73**				
**74**				
**74**				Antiaggregatory, cytoprotective, antiparkinsonian

^1^ Qualitative bioassay study reported without quantitative data was marked with O.

**Table 8:** Reported biological activities of compounds **76–89**.

No.	Biological Activities ^1^							
	Antioxidant		Antidiabetic	Cytotoxic		Anti-Inflammatory	Antibacterial	Others
	**DPPH Scavenging IC_50_ (μM)**	**Superoxide Radical Scavenging IC_50_ (μM)**	**AGE Production Inhibitory IC_50_ (μM)**	**HepG2 EC_50_ (μM)**	**A549 EC_50_ (μM)**			
**76**	O	O	O			O	O	Anticancer, anti-ulcerantiaging, antifibrotic, antiviral
**77**	O	O				O		Anti-microbial, anticancer
**78**	O	O	O			O	O	Antiviral, anti-atherosclerotic, immunostimulatory, cardioprotective, antiproliferative, hepatoprotective, anticancer, antihepatocarcinoma
**79**								
**80**						O		
**81**	O			78.5		O		Antimicrobial, apoptosis- and autophagy-modulating, anxiolytic, anticonvulsant, immunomodulatory, antinociceptive
**82**	27.6	38.6		56.3	48.9			
**83**	O			O			O	Melanogenesis-inhibitory
**84**								Antimicrobial, α-glucosidase inhibition, cathepsin B inhibition
**85**	O							Antidepressant
**86**	O			O				Hepatoprotective, acetylcholinesterase, hypouricemic, antifungal, immunomoudlatory, antithyroid, anti-P-388 murine leukemia cell, hypoglycemic, hypolipidemic, antiaging
**87**				17.6	6.2			
**88**				O				Anti-plasmodial
**89**	O							

^1^ Qualitative bioassay study reported without quantitative data was marked with O.

**Table 9:** Yield of the compounds.

Compound No.	Yield ^2^ (ppm)	Note ^1^ and Reference No.	Compound No.	YieldN ^2^ (ppm)	Note ^1^ and Reference No.	Compound No.	Yield ^2^ (ppm)	Note ^1^ and Reference No.
**1**	123.9	Dried [16]	**31**	0.2	Wet [77]	**61**	0.2	Wet [36]
**2**	N.A.		**32**	0.3		**62**	0.12	
**3**	N.A.		**33**	1.3		**63**	0.2	Wet [120]
**4**	N.A.		**34**	0.2		**64**	0.2	
**5**	0.1	Wet [19]	**35**	0.4		**65**	0.3	
**6**	0.2		**36**	1.6	Wet [80]	**66**	N.A.	
**7**	0.1		**37**	0.9		**67**	0.2	Wet [36]
**8**	0.1		**38**	0.4		**68**	3.8	Wet [130]
**9**	0.1		**39**	700	Dried [81]	**69**	0.3	Wet [111]
**10**	0.6	Wet [32]	**40**	93.3		**70**	0.5	
**11**	0.5		**41**	466.7		**71**	0.6	
**12**	0.3		**42**	33.3		**72**	0.2	
**13**	0.4		**43**	41.7		**73**	0.1	Wet [36]
**14**	0.1	Wet [36]	**44**	0.5	Wet [6]	**74**	0.1	
**15**	0.1		**45**	0.9	Dried [95]	**75**	0.1	
**16**	0.1		**46**	0.6		**76**	1.54	Dried [135]
**17**	0.1		**47**	0.7		**77**	8.54	
**18**	0.1		**48**	0.1	Wet [36]	**78**	6.87	
**19**	0.2		**49**	0.3		**79**	1.3	Dried [108]
**20**	0.1		**50**	0.1		**80**	0.9	Wet [120]
**21**	0.1		**51**	N.A.		**81**	0.2	Wet [111]
**22**	8	Dried [50]	**52**	0.3	Wet [105]	**82**	1.2	
**23**	6.2	Wet [6]	**53**	0.4		**83**	0.3	
**24**	0.1		**54**	0.5		**84**	0.2	
**25**	0.4		**55**	0.3		**85**	0.5	
**26**	0.6		**56**	0.4	Dried [95]	**86**	0.2	
**27**	16.3	Dried [75]	**57**	0.4		**87**	1.1	Wet [32]
**28**	1.4		**58**	20.7	Dried [108]	**88**	0.6	
**29**	12.8		**59**	9.7		**89**	0.9	
**30**	1.7		**60**	0.3	Wet [111]			

^1^ Plant material status before extraction. Dried or Wet. ^2^ data Not Available (N.A.).

## References

[1] Singh D., Buhmann A.K., Flowers T.J., Seal C.E., Papenbrock J. (2014). Salicornia as a crop plant in temperate regions: Selection of genetically characterized ecotypes and optimization of their cultivation conditions. AoB Plants.

[2] Yamamoto K., Oguri S., Chiba S., Momonoki Y.S. (2009). Molecular cloning of acetylcholinesterase gene from *Salicornia europaea* L.. Plant Signal. Behav..

[3] Kim C.S., Song T.G. (1983). Ecological Studies on the Halophyte Communities at Western and Southern Coasts in Korea (IV). Korean J. Ecol..

[4] Rhee M.H., Park H.J., Cho J.Y. (2009). Salicornia herbacea: Botanical, chemical and pharmacological review of halophyte marsh plant. J. Med. Plants Res..

[5] Kim Y.A., Kong C.S., Lee J.I., Kim H., Park H.Y., Lee H.S., Lee C., Seo Y. (2012). Evaluation of novel antioxidant triterpenoid saponins from the halophyte Salicornia herbacea. Bioorganic Med. Chem. Lett..

[6] Kim J.Y., Cho J.Y., Ma Y.K., Park K.Y., Lee S.H., Ham K.S., Lee H.J., Park K.H., Moon J.H. (2011). Dicaffeoylquinic acid derivatives and flavonoid glucosides from glasswort (*Salicornia herbacea* L.) and their antioxidative activity. Food Chem..

[7] Patel S. (2016). Salicornia: Evaluating the halophytic extremophile as a food and a pharmaceutical candidate. 3 Biotech.

[8] Im S.-A., Kim G.-W., Lee C.-K. (2003). Immunomodulatory Activity of *Salicornia herbacea* L. Components. Nat. Prod. Sci..

[9] Lee J.M., Yim M.J., Choi G., Lee M.S., Park Y.G., Lee D.S. (2018). Antioxidant and anti-inflammatory activity of six halophytes in Korea. Nat. Prod. Sci..

[10] Park S.H., Ko S.K., Choi J.G., Chung S.H. (2006). Salicornia herbacea prevents high fat diet-induced hyperglycemia and hyperlipidemia in ICR mice. Arch. Pharm. Res..

[11] Lee K.Y., Lee M.H., Chang I.Y., Yoon S.P., Lim D.Y., Jeon Y.J. (2006). Macrophage activation by polysaccharide fraction isolated from Salicornia herbacea. J. Ethnopharmacol..

[12] Favel A., Steininetz M.D., Regli P., Vidal-Ollivier E., Flias R., Balansard G. (1994). In Vitro Antifungal Activity of Triterpenoid Saponins. Planta Med..

[13] Simões C.M.O., Amoros M., Girre L. (1999). Mechanism of antiviral activity of triterpenoid saponins. Phyther. Res..

[14] Xi M., Hai C., Tang H., Wen A., Chen H., Liu R., Liang X., Chen M. (2010). Antioxidant and antiglycation properties of triterpenoid saponins from Aralia taibaiensis traditionally used for treating diabetes mellitus. Redox Rep..

[15] Jia L.Y., Wu X.J., Gao Y., Rankin G.O., Pigliacampi A., Bucur H., Li B., Tu Y.Y., Chen Y.C. (2017). Inhibitory effects of total triterpenoid saponins isolated from the seeds of the tea plant (camellia sinensis) on human ovarian cancer cells. Molecules.

[16] Shan Y., Huan L., Fuqin G., Chen Y., Min Y., Wang M., Feng X., Wang Q. (2015). Triterpenoids from the herbs of salicornia bigelovii. Molecules.

[17] Braut-Boucher F., Achard-Ellouk S., Pauthe-Dayde D., Henry M., Hoellinger H. (1990). Cytoprotective effects of Gypsophila saponins towards isolated rat hepatocytes. Food Addit. Contam..

[18] Li F., Li W., Fu H., Zhang Q., Koike K. (2007). Pancreatic lipase-inhibiting triterpenoid saponins from fruits of Acanthopanax senticosus. Chem. Pharm. Bull..

[19] Yin M., Wang X., Wang M., Chen Y., Dong Y., Zhao Y., Feng X. (2012). A new triterpenoid saponin and other saponins from Salicornia europaea. Chem. Nat. Compd..

[20] Chiozem D.D., Trinh-Van-Dufat H., Wansi J.D., Mbazoa Djama C., Fannang V.S., Seguin E., Tillequin F., Wandji J. (2009). New friedelane triterpenoids with antimicrobial activity from the stems of Drypetes paxii. Chem. Pharm. Bull..

[21] Kang O.-H., Kang S.-H., Kim S.-B., Mun S.-H., Seo Y.-S., Joung D.-K., Kim M.-R., Shin D.-W., Kweon K.-T., Kwon D.-Y. (2012). Anti-inflammatory effect of oleanoic acid 28-O-β-D-glycopyranosyl ester isolated from Aralia cordata in activated HMC-1 cells. Afr. J. Pharm. Pharmacol..

[22] Guo T., Wu S., Guo S., Bai L., Liu Q., Bai N. (2015). Synthesis and Evaluation of a Series of Oleanolic Acid Saponins as α-Glucosidase and α-Amylase Inhibitors. Arch. Pharm..

[23] Wang J., Lu J., Lv C., Xu T., Jia L. (2012). Three new triterpenoid saponins from root of Gardenia jasminoides Ellis. Fitoterapia.

[24] Lee K.M., Yun J.H., Lee D.H., Park Y.G., Son K.H., Nho C.W., Kim Y.S. (2015). Chikusetsusaponin IVa methyl ester induces cell cycle arrest by the inhibition of nuclear translocation of β-catenin in HCT116 cells. Biochem. Biophys. Res. Commun..

[25] Chen X., Wu Q.S., Meng F.C., Tang Z.H., Chen X., Lin L.G., Chen P., Qiang W.A., Wang Y.T., Zhang Q.W. (2016). Chikusetsusaponin IVa methyl ester induces G1 cell cycle arrest, triggers apoptosis and inhibits migration and invasion in ovarian cancer cells. Phytomedicine.

[26] Lee H.J., Shin J.S., Lee W.S., Shim H.Y., Park J.M., Jang D.S., Lee K.T. (2016). Chikusetsusaponin iva methyl ester isolated from the roots of achyranthes japonica suppresses LPS-Induced iNOS, TNF-α, IL-6, and IL-1β Expression by NF-eκB and AP-1 Inactivation. Biol. Pharm. Bull..

[27] Das N., Chandran P., Chakraborty S. (2011). Potent spermicidal effect of oleanolic acid 3-beta-d-glucuronide, an active principle isolated from the plant Sesbania sesban Merrill. Contraception.

[28] Guan F., Wang Q., Wang M., Shan Y., Chen Y., Yin M., Zhao Y., Feng X., Liu F., Zhang J. (2015). Isolation, identification and cytotoxicity of a new noroleanane-type triterpene saponin from Salicornia bigelovii Torr. Molecules.

[29] Lee B., Lee D.Y., Yoo K.H., Baek N.I., Park J.H., Chung I.S. (2012). Calenduloside E 6′-methyl ester induces apoptosis in CT-26 mouse colon carcinoma cells and inhibits tumor growth in a CT-26 xenograft animal model. Oncol. Lett..

[30] Wang Q.Z., Liu X.F., Shan Y., Guan F.Q., Chen Y., Wang X.Y., Wang M., Feng X. (2012). Two new nortriterpenoid saponins from Salicornia bigelovii Torr. and their cytotoxic activity. Fitoterapia.

[31] Yang B., Zhu J.P., Rong L., Jin J., Cao D., Li H., Zhou X.H., Zhao Z.X. (2018). Triterpenoids with antiplatelet aggregation activity from Ilex rotunda. Phytochemistry.

[32] Zhao Y., Wang X., Wang H., Liu T., Xin Z. (2014). Two new noroleanane-type triterpene saponins from the methanol extract of Salicornia herbacea. Food Chem..

[33] Ayeleso T.B., Matumba M.G., Mukwevho E. (2017). Oleanolic acid and its derivatives: Biological activities and therapeutic potential in chronic diseases. Molecules.

[34] Khwaza V., Oyedeji O.O., Aderibigbe B.A. (2018). Antiviral activities of oleanolic acid and its analogues. Molecules.

[35] Emirdaǧ-Öztürk S., Karayildirim T., Çapci-Karagöz A., Alankuş-Çalişkan Ö., Özmen A., Poyrazoǧlu-Çoban E. (2014). Synthesis, antimicrobial and cytotoxic activities, and structure-activity relationships of gypsogenin derivatives against human cancer cells. Eur. J. Med. Chem..

[36] Lyu H., Ma X., Guan F., Chen Y., Wang Q., Feng X. (2018). 30-Noroleanane triterpenoid saponins from Salicornia europaea Linn. and their chemotaxonomic significance. Biochem. Syst. Ecol..

[37] Wei Y., Ma C.M., Chen D.Y., Hattori M. (2008). Anti-HIV-1 protease triterpenoids from Stauntonia obovatifoliola Hayata subsp. intermedia. Phytochemistry.

[38] Wang J., Xu Q.L., Zheng M.F., Ren H., Lei T., Wu P., Zhou Z.Y., Wei X.Y., Tan J.W. (2014). Bioactive 30-noroleanane triterpenes from the pericarps of akebia trifoliata. Molecules.

[39] Chowdhury M.A., Ko H.J., Lee H., Aminul Haque M., Park I.S., Lee D.S., Woo E.R. (2017). Oleanane triterpenoids from Akebiae Caulis exhibit inhibitory effects on Aβ42 induced fibrillogenesis. Arch. Pharm. Res..

[40] Ouyang J.K., Dong L.M., Xu Q.L., Wang J., Liu S.B., Qian T., Yuan Y.F., Tan J.W. (2018). Triterpenoids with α-glucosidase inhibitory activity and cytotoxic activity from the leaves of Akebia trifoliata. RSC Adv..

[41] Espada A., Rodriguez J., Villaverde M.C., Riguera R. (1990). Hypoglucaemic triterpenoid saponins from Boussingaultia baselloides. Can. J. Chem..

[42] Fang J.B., Yao Z., Chen J.C., Liu Y.W., Takaishi Y., Duan H.Q. (2009). Cytotoxic triterpene saponins from alternanthera philoxeroides. J. Asian Nat. Prod. Res..

[43] Thiyagarajan G., Muthukumaran P., Sarath Kumar B., Muthusamy V.S., Lakshmi B.S. (2016). Selective Inhibition of PTP1B by Vitalboside A from Syzygium cumini Enhances Insulin Sensitivity and Attenuates Lipid Accumulation Via Partial Agonism to PPARγ: In Vitro and In Silico Investigation. Chem. Biol. Drug Des..

[44] Rattanathongkom A., Lee J.B., Hayashi K., Sripanidkulchai B.O., Kanchanapoom T., Hayashi T. (2009). Evaluation of chikusetsusaponin IVa isolated from Alternanthera philoxeroides for its potency against viral replication. Planta Med..

[45] Dahmer T., Berger M., Barlette A.G., Reck J., Segalin J., Verza S., Ortega G.G., Gnoatto S.C.B., Guimarães J.A., Verli H. (2012). Antithrombotic effect of chikusetsusaponin IVa isolated from Ilex paraguariensis (Maté). J. Med. Food.

[46] Cui J., Xi M.M., Li Y.W., Duan J.L., Wang L., Weng Y., Jia N., Cao S.S., Li R.L., Wang C. (2015). Insulinotropic effect of Chikusetsu saponin IVa in diabetic rats and pancreatic β-cells. J. Ethnopharmacol..

[47] Wang H., Qi J., Li L., Wu T., Wang Y., Wang X., Ning Q. (2015). Inhibitory effects of Chikusetsusaponin IVa on lipopolysaccharide-induced proinflammatory responses in THP-1 cells. Int. J. Immunopathol. Pharmacol..

[48] Yin J., Seo C.S., Hwang I.H., Lee M.W., Song K.H. (2018). Anti-obesity activities of chikusetsusaponin IVa and Dolichos lablab L. Seeds. Nutrients.

[49] Miyamae Y., Kurisu M., Han J., Isoda H., Shigemori H. (2011). Structure-activity relationship of caffeoylquinic acids on the accelerating activity on ATP production. Chem. Pharm. Bull..

[50] Chung Y.C., Chun H.K., Yang J.Y., Kim J.Y., Han E.H., Kho Y.H., Jeong H.G. (2005). Tungtungmadic acid, a novel antioxidant, from Salicornia herbacea. Arch. Pharm. Res..

[51] Hwang Y.P., Yun H.J., Chun H.K., Chung Y.C., Kim H.K., Jeong M.H., Yoon T.R., Jeong H.G. (2009). Protective mechanisms of 3-caffeoyl, 4-dihydrocaffeoyl quinic acid from Salicornia herbacea against tert-butyl hydroperoxide-induced oxidative damage. Chem. Biol. Interact..

[52] Han E.H., Kim J.Y., Kim H.G., Chun H.K., Chung Y.C., Jeong H.G. (2010). Inhibitory effect of 3-caffeoyl-4-dicaffeoylquinic acid from Salicornia herbacea against phorbol ester-induced cyclooxygenase-2 expression in macrophages. Chem. Biol. Interact..

[53] Hwang Y.P., Yun H.J., Choi J.H., Chun H.K., Chung Y.C., Kim S.K., Kim B.H., Kwon K.I., Jeong T.C., Lee K.Y. (2010). 3-Caffeoyl, 4-dihydrocaffeoylquinic acid from Salicornia herbacea inhibits tumor cell invasion by regulating protein kinase C-δ-dependent matrix metalloproteinase-9 expression. Toxicol. Lett..

[54] Hwang Y.P., Kim H.G., Choi J.H., Do M.T., Tran T.P., Chun H.K., Chung Y.C., Jeong T.C., Gwang J.H. (2013). 3-Caffeoyl, 4-dihydrocaffeoylquinic acid from Salicornia herbacea attenuates high glucose-induced hepatic lipogenesis in human HepG2 cells through activation of the liver kinase B1 and silent information regulator T1/AMPK-dependent pathway. Mol. Nutr. Food Res..

[55] Ooi L.S.M., Wang H., He Z., Ooi V.E.C. (2006). Antiviral activities of purified compounds from Youngia japonica (L.) DC (Asteraceae, Compositae). J. Ethnopharmacol..

[56] Zhang M., Liu W.X., Zheng M.F., Xu Q.L., Wan F.H., Wang J., Lei T., Zhou Z.Y., Tan J.W. (2013). Bioactive quinic acid derivatives from ageratina adenophora. Molecules.

[57] Gray N.E., Morre J., Kelley J., Maier C.S., Stevens J.F., Quinn J., Soumaynath A. (2014). Caffeoylquinic Acids in Centella asiatica Protect Against β-amyloid toxicity. J. Alzheimer’s Dis..

[58] Kim J.Y., Lee H.K., Hwang B.Y., Kim S.H., Yoo J.K., Seong Y.H. (2012). Neuroprotection of ilex latifolia and caffeoylquinic acid derivatives against excitotoxic and hypoxic damage of cultured rat cortical neurons. Arch. Pharm. Res..

[59] Nurul Islam M., Jung H.A., Sohn H.S., Kim H.M., Choi J.S. (2013). Potent α-glucosidase and protein tyrosine phosphatase 1B inhibitors from Artemisia capillaris. Arch. Pharm. Res..

[60] Chen J., Mangelinckx S., Ma L., Wang Z., Li W., De Kimpe N. (2014). Caffeoylquinic acid derivatives isolated from the aerial parts of Gynura divaricata and their yeast α-glucosidase and PTP1B inhibitory activity. Fitoterapia.

[61] Lee Y.K., Hong E.Y., Whang W.K. (2017). Inhibitory Effect of Chemical Constituents Isolated from Artemisia iwayomogi on Polyol Pathway and Simultaneous Quantification of Major Bioactive Compounds. Biomed Res. Int..

[62] Yoon M.H., Cho C.W., Lee J.W., Kim Y.S., An G.H., Lim C.H. (2008). Antithrombotic compounds from the Leaves of Ligularia stenocephala M. Nat. Prod. Sci..

[63] Park K.H., Park M., Choi S.E., Jeong M.S., Kwon J.H., Oh M.H., Choi H.K., Seo S.J., Lee M.W. (2009). The anti-oxidative and anti-inflammatory effects of caffeoyl derivatives from the roots of Aconitum koreanum R. Raymond. Biol. Pharm. Bull..

[64] Hao B.J., Wu Y.H., Wang J.G., Hu S.Q., Keil D.J., Hu H.J., Lou J.D., Zhao Y. (2012). Hepatoprotective and antiviral properties of isochlorogenic acid A from Laggera alata against hepatitis B virus infection. J. Ethnopharmacol..

[65] Zhao Y., Geng C.A., Ma Y.B., Huang X.Y., Chen H., Cao T.W., He K., Wang H., Zhang X.M., Chen J.J. (2014). UFLC/MS-IT-TOF guided isolation of anti-HBV active chlorogenic acid analogues from Artemisia capillaris as a traditional Chinese herb for the treatment of hepatitis. J. Ethnopharmacol..

[66] Heyman H.M., Senejoux F., Seibert I., Klimkait T., Maharaj V.J., Meyer J.J.M. (2015). Identification of anti-HIV active dicaffeoylquinic- and tricaffeoylquinic acids in Helichrysum populifolium by NMR-based metabolomic guided fractionation. Fitoterapia.

[67] Fan L., Wang Y., Liang N., Huang X.J., Fan C.L., Wu Z.L., He Z.D., Li Y.L., Ye W.C. (2015). Quinic acid derivatives and coumarin glycoside from the roots and stems of Erycibe obtusifolia. Phytochem. Lett..

[68] Teoh W.Y., Wahab N.A., Sim K.S. (2018). Caffeoylquinic acids induce cell death and cell cycle arrest on HCT 116 cells via formation of extracellular H2O2and quinones. Chiang Mai J. Sci..

[69] Lee S.Y., Moon E., Kim S.Y., Lee K.R. (2013). Quinic acid derivatives from Pimpinella brachycarpa exert anti-neuroinflammatory activity in lipopolysaccharide-induced microglia. Bioorganic Med. Chem. Lett..

[70] Kim J.Y., Lee H.K., Seong Y.H. (2019). Anti-nociceptive and anti-inflammatory properties of ilex latifolia and its active component, 3,5-di-caffeoyl quinic acid methyl ester. Nat. Prod. Sci..

[71] Hu W., Shen T., Wang M.H. (2011). Cell cycle arrest and apoptosis induced by methyl 3,5-dicaffeoyl quinate in human colon cancer cells: Involvement of the PI3K/Akt and MAP kinase pathways. Chem. Biol. Interact..

[72] Hu T., He X.W., Jiang J.G. (2014). Functional analyses on antioxidant, anti-inflammatory, and antiproliferative effects of extracts and compounds from Ilex latifolia Thunb., a Chinese bitter tea. J. Agric. Food Chem..

[73] Shen T., Heo S.I., Wang M.H. (2012). Involvement of the p38 MAPK and ERK signaling pathway in the anti-melanogenic effect of methyl 3,5-dicaffeoyl quinate in B16F10 mouse melanoma cells. Chem. Biol. Interact..

[74] Liu H., Zhang X., Wu C., Wu H., Guo P., Xu X. (2013). Anti-hyperlipidemic caffeoylquinic acids from the fruits of pandanustectorius soland. J. Appl. Pharm. Sci..

[75] Tuan N.Q., Lee W., Oh J., Kwak S., Lee H.G., Ferreira D., Bae J.S., Na M.K. (2015). Quinic acid derivatives from Salicornia herbacea alleviate HMGB1-mediated endothelial dysfunction. J. Funct. Foods.

[76] Li X., Li K., Xie H., Xie Y., Li Y., Zhao X., Jiang X., Chen D. (2018). Antioxidant and cytoprotective effects of the Di-O-Caffeoylquinic acid family: The mechanism, structure–activity relationship, and conformational effect. Molecules.

[77] Cho J.Y., Kim J.Y., Lee Y.G., Lee H.J., Shim H.J., Lee J.H., Kim S.J., Ham K.S., Moon J.H. (2016). Four new dicaffeoylquinic acid derivatives from glasswort (*Salicornia herbacea* L.) and their antioxidative activity. Molecules.

[78] Kashiwada Y., Ahmed F.A., Kurimoto S.I., Kim S.Y., Shibata H., Fujioka T., Takaishi Y. (2012). New α-glucosides of caffeoyl quinic acid from the leaves of Moringa oleifera Lam. J. Nat. Med..

[79] Kim M., Choi S.Y., Lee P., Hur J. (2015). Neochlorogenic Acid Inhibits Lipopolysaccharide-Induced Activation and Pro-inflammatory Responses in BV2 Microglial Cells. Neurochem. Res..

[80] Arakawa Y., Asada Y., Ishida H. (1982). Instructions for use Structures of New Two Isoflavones and One Flavanone from Glasswort (*Salicornia europaea* L.). J. Fac. Agr. Hokkaido Univ..

[81] Geslin M., Verbist J.F. (1985). Flavonoides de salicornia europaea. J. Nat. Prod..

[82] Shimoda H., Nakamura S., Morioka M., Tanaka J., Matsuda H., Yoshikawa M. (2011). Effect of cinnamoyl and flavonol glucosides derived from cherry blossom flowers on the production of advanced glycation end products (AGEs) and AGE-induced fibroblast apoptosis. Phyther. Res..

[83] Azuma K., Nakayama M., Koshioka M., Ippoushi K., Yamaguchi Y., Kohata K., Yamauchi Y., Ito H., Higashio H. (1999). Phenolic antioxidants from the leaves of *Corchorus olitorius* L.. J. Agric. Food Chem..

[84] Jaramillo K., Dawid C., Hofmann T., Fujimoto Y., Osorio C. (2011). Identification of antioxidative flavonols and anthocyanins in Sicana odorifera fruit peel. J. Agric. Food Chem..

[85] Anand David A.V., Arulmoli R., Parasuraman S. (2016). Overviews of biological importance of quercetin: A bioactive flavonoid. Pharmacogn. Rev..

[86] Valentová K., Vrba J., Bancířová M., Ulrichová J., Křen V. (2014). Isoquercitrin: Pharmacology, toxicology, and metabolism. Food Chem. Toxicol..

[87] Ganeshpurkar A., Saluja A.K. (2017). The Pharmacological Potential of Rutin. Saudi Pharm. J..

[88] Lee Y.S., Lee S., Lee H.S., Kim B.K., Ohuchi K., Shin K.H. (2005). Inhibitory effects of isorhamnetin-3-O-β-D-glucoside from salicornia herbacea on rat lens aldose reductase and sorbitol accumulation in streptozotocin-induced diabetic rat tissues. Biol. Pharm. Bull..

[89] Kong C.S., Kim Y.A., Kim M.M., Park J.S., Kim J.A., Kim S.K., Lee B.J., Nam T.J., Seo Y. (2008). Flavonoid glycosides isolated from Salicornia herbacea inhibit matrix metalloproteinase in HT1080 cells. Toxicol. Vitr..

[90] Kong C.S., Kim J.A., Qian Z.J., Kim Y.A., Lee J.I., Kim S.K., Nam T.J., Seo Y. (2009). Protective effect of isorhamnetin 3-O{cyrillic}-β-d-glucopyranoside from Salicornia herbacea against oxidation-induced cell damage. Food Chem. Toxicol..

[91] Kong C.S., Lee J.I., Kim Y.A., Kim J.A., Bak S.S., Hong J.W., Park H.Y., Yea S.S., Seo Y. (2012). Evaluation on anti-adipogenic activity of flavonoid glucopyranosides from Salicornia herbacea. Process Biochem..

[92] Kim K.-S., Park S.-H. (2004). Isolation and Identification of Antioxidant Flavonoids from *Salicornia herbacea* L.. Appl. Biol. Chem..

[93] Rice-Evans C.A., Miller N.J., Paganga G. (1996). Structure-Antioxidant Activity Relationships of Flavonoids and Phenolic Acids. Free Radic. Biol. Med..

[94] Burda S., Oleszek W. (2001). Antioxidant and antiradical activities of flavonoids. J. Agric. Food Chem..

[95] Tuan N.Q., Lee W., Oh J., Kulkarni R.R., Gény C., Jung B., Kang H., Bae J.S., Na M.K. (2015). Flavanones and Chromones from Salicornia herbacea Mitigate Septic Lethality via Restoration of Vascular Barrier Integrity. J. Agric. Food Chem..

[96] Mosihuzzman M., Naheed S., Hareem S., Talib S., Abbas G., Khan S.N., Choudhary M.I., Sener B., Tareen R.B., Israr M. (2013). Studies on α-glucosidase inhibition and anti-glycation potential of Iris loczyi and Iris unguicularis. Life Sci..

[97] Luo Y., Shang P., Li D. (2017). Luteolin: A Flavonoid that has multiple cardio-protective effects and its molecular mechanisms. Front. Pharmacol..

[98] Calderón-Montaño J.M., Burgos-Morón E., Pérez-Guerrero C., López-Lázaro M. (2011). A Review on the Dietary Flavonoid Kaempferol | BenthamScience. Mini Rev. Med. Chem..

[99] Riaz A., Rasul A., Hussain G., Zahoor M.K., Jabeen F., Subhani Z., Younis T., Ali M., Sarfraz I., Selamoglu Z. (2018). Astragalin: A Bioactive Phytochemical with Potential Therapeutic Activities. Adv. Pharmacol. Sci..

[100] Kim J., Karthivashan G., Kweon M.H., Kim D.H., Choi D.K. (2019). The Ameliorative Effects of the Ethyl Acetate Extract of Salicornia europaea L. and Its Bioactive Candidate, Irilin B, on LPS-Induced Microglial Inflammation and MPTP-Intoxicated PD-Like Mouse Model. Oxid. Med. Cell. Longev..

[101] Bergeron C., Marston A., Hakizamungu E., Hostettmann K. (1995). Antifungal constituents of chenopodium procerum. Pharm. Biol..

[102] Moein M.R., Khan S.I., Ali Z., Ayatollahi S.A.M., Kobarfard F., Nasim S., Choudhary M.I., Khan I.A. (2008). Flavonoids from Iris songarica and their antioxidant and estrogenic activity. Planta Med..

[103] Gaspar A., Matos M.J., Garrido J., Uriarte E., Borges F. (2014). Chromone: A valid scaffold in medicinal chemistry. Chem. Rev..

[104] Reis J., Gaspar A., Milhazes N., Borges F. (2017). Chromone as a Privileged Scaffold in Drug Discovery: Recent Advances. J. Med. Chem..

[105] Chiji H., Izawa M., Aiba T. (1978). Isolation and Identification of Two 2,3-Unsubstituted Chromones from Glasswort (*Salicornia europaea* L.). Agric. Biol. Chem..

[106] Arakawa Y., Chiji H., Izawa M. (1983). Structural Elucidation of Two New Chromones Isolated from Glasswort (*Salicornia europaea* L.). Agric. Biol. Chem..

[107] Hartmann M.A. (1998). Plant sterols and the membrane environment. Trends Plant Sci..

[108] Lee Y.S., Hye S.L., Kuk H.S., Kim B.K., Lee S. (2004). Constituents of the halophyte Salicornia herbacea. Arch. Pharm. Res..

[109] Saeidnia S. (2014). The Story of Beta-sitosterol- A Review. Eur. J. Med. Plants.

[110] Kaur N., Chaudhary J., Jain A., Kishore L. (2011). Stigmasterol: A comprehensive Review. Int. J. Pharm. Sci. Res..

[111] Wang X., Zhang M., Zhao Y., Wang H., Liu T., Xin Z. (2013). Pentadecyl ferulate, a potent antioxidant and antiproliferative agent from the halophyte Salicornia herbacea. Food Chem..

[112] Kim J.A., Tay D., de Blanco E.C. (2008). NF-κB Inhibitory Activity of Compounds isolated from Cantharellus cibarius. Phyther. Res..

[113] Kawagishi H., Katsumi R., Sazawa T., Mizuno T., Hagiwara T., Nakamura T. (1988). Cytotoxic Steroids from the Mushroom Agaricus Blazei. Phytochemistry.

[114] Appiah T., Agyare C., Luo Y., Boamah V.E., Boakye Y.D. (2018). Antimicrobial and Resistance Modifying Activities of Cerevisterol Isolated from Trametes Species. Curr. Bioact. Compd..

[115] Lee J.H., Lee J.Y., Park J.H., Jung H.S., Kim J.S., Kang S.S., Kim Y.S., Han Y. (2007). Immunoregulatory activity by daucosterol, a β-sitosterol glycoside, induces protective Th1 immune response against disseminated Candidiasis in mice. Vaccine.

[116] Choi J.N., Choi Y.H., Lee J.M., Noh I.C., Park J.W., Choi W.S., Choi J.H. (2012). Anti-inflammatory effects of β-sitosterol-β-D-glucoside from Trachelospermum jasminoides (Apocynaceae) in lipopolysaccharide-stimulated RAW 264.7 murine macrophages. Nat. Prod. Res..

[117] Zhao C., She T., Wang L., Su Y., Qu L., Gao Y., Xu S., Cai S., Shou C. (2015). Daucosterol inhibits cancer cell proliferation by inducing autophagy through reactive oxygen species-dependent manner. Life Sci..

[118] Jiang L.H., Yang N.Y., Yuan X.L., Zou Y.J., Zhao F.M., Chen J.P., Wang M.Y., Lu D.X. (2014). Daucosterol promotes the proliferation of neural stem cells. J. Steroid Biochem. Mol. Biol..

[119] Saleem M., Hyoung J.K., Ali M.S., Yong S.L. (2005). An update on bioactive plant lignans. Nat. Prod. Rep..

[120] Wang X., Feng X., Wang M., Chen Y., Dong Y., Zhao Y., Sun H. (2011). Studies on the chemical constituents of Salicornia europaea. Zhong Yao Cai.

[121] Jung M.J., Kang S.S., Jung H.A., Kim G.J., Choi J.S. (2004). Isolation of flavonoids and a cerebroside from the stem bark of Albizzia julibrissin. Arch. Pharm. Res..

[122] Luecha P., Umehara K., Miyase T., Noguchi H. (2009). Antiestrogenic constituents of the Thai medicinal plants Capparis flavicans and Vitex glabrata. J. Nat. Prod..

[123] Wei Q., Qiu Z., Xu F., Li Q., Yin H. (2015). Chemical Components from Leaves of Fatisai Japonica and Their Antitumor Activities in Vitro. Zhong Yao Cai.

[124] Wang S., Wu C., Li X., Zhou Y., Zhang Q., Ma F., Wei J., Zhang X., Guo P. (2017). Syringaresinol-4-O-β-D-glucoside alters lipid and glucose metabolism in HepG2 cells and C2C12 myotubes. Acta Pharm. Sin. B.

[125] Yang Y.P., Cheng M.J., Teng C.M., Chang Y.L., Tsai I.L., Chen I.S. (2002). Chemical and anti-platelet constituents from Formosan Zanthoxylum simulans. Phytochemistry.

[126] Wang L.Y., Unehara N., Kitanaka S. (2005). Lignans from the Roots of Wikstroemia indica and their DPPH radical scavenging and nitric oxide inhibitory activities. Chem. Pharm. Bull..

[127] Jeong Y.H., Chung S.Y., Han A.R., Sung M.K., Jang D.S., Lee J., Kwon Y., Lee H.J., Seo E.K. (2007). P-glycoprotein inhibitory activity of two phenolic compounds, (-)-syringaresinol and tricin from Sasa borealis. Chem. Biodivers..

[128] Oh J.H., Joo Y.H., Karadeniz F., Ko J., Kong C.S. (2020). Syringaresinol inhibits UVA-induced MMP-1 expression by suppression of mapk/ap-1 signaling in hacat keratinocytes and human dermal fibroblasts. Int. J. Mol. Sci..

[129] Wang W., Li N., Wang J., Chen G., Huang R., Zhao W., Li J., Si Y. (2016). Bioactive benzofuran-chalcanes as potential NQO1 inducers from Millettia pulchra (Benth) kurzvar-laxior (Dunn) Z.Wei. Phytochemistry.

[130] Karthivashan G., Kweon M.H., Park S.Y., Kim J.S., Kim D.H., Ganesan P., Choi D.K. (2019). Cognitive-enhancing and ameliorative effects of acanthoside B in a scopolamine-induced amnesic mouse model through regulation of oxidative/inflammatory/cholinergic systems and activation of the TrkB/CREB/BDNF pathway. Food Chem. Toxicol..

[131] Kapoor R., Huang Y.-S. (2006). Gamma Linolenic Acid: An Antiinflammatory Omega-6 Fatty Acid. Curr. Pharm. Biotechnol..

[132] Taylor J.C., Rapport L., Lockwood G.B. (2003). Octacosanol in human health. Nutrition.

[133] Wang T., Liu Y.Y., Wang X., Yang N., Zhu H.B., Zuo P.P. (2010). Protective effects of octacosanol on 6-hydroxydopamine-induced Parkinsonism in rats via regulation of ProNGF and NGF signaling. Acta Pharmacol. Sin..

[134] Kaushik M.K., Aritake K., Takeuchi A., Yanagisawa M., Urade Y. (2017). Octacosanol restores stress-affected sleep in mice by alleviating stress. Sci. Rep..

[135] Oh J.H., Kim E.O., Lee S.K., Woo M.H., Choi S.W. (2007). Antioxidant activities of the ethanol extract of Hamcho (*Salicornia herbacea* L.) cake prepared by enzymatic treatment. Food Sci. Biotechnol..

[136] Kakkar S., Bais S. (2014). A Review on Protocatechuic Acid and Its Pharmacological Potential. ISRN Pharmacol..

[137] Ou S., Kwok K.C. (2004). Ferulic acid: Pharmaceutical functions, preparation and applications in foods. J. Sci. Food Agric..

[138] Monteiro Espíndola K.M., Ferreira R.G., Mosquera Narvaez L.E., Rocha Silva Rosario A.C., Machado Da Silva A.H., Bispo Silva A.G., Oliveira Vieira A.P., Chagas Monteiro M. (2019). Chemical and pharmacological aspects of caffeic acid and its activity in hepatocarcinoma. Front. Oncol..

[139] Zhou Y., Jin M., Jin C., Ye C., Wang J., Wang R., Wei C., Zhou W., Li G. (2019). Megastigmane derivatives from Corispermum mongolicum and their anti-inflammatory activities. Phytochem. Lett..

[140] Islam M.T., Ali E.S., Uddin S.J., Shaw S., Islam M.A., Ahmed M.I., Chandra Shill M., Karmakar U.K., Yarla N.S., Khan I.N. (2018). Phytol: A review of biomedical activities. Food Chem. Toxicol..

[141] Sastry V.M.V.S., Rao G.R.K. (1995). Dioctyl phthalate, and antibacterial compound from the marine brown alga—Sargassum wightii. J. Appl. Phycol..

[142] Nguyen D.T.M., Nguyen D.H., La Lyun H., Lee H.B., Shin J.H., Kim E.K. (2007). Inhibition of melanogenesis by dioctyl phthalate isolated from Nigella glandulifera Freyn. J. Microbiol. Biotechnol..

[143] Aboul-Enein A.M., Shanab S.M.M., Shalaby E.A., Zahran M.M., Lightfoot D.A., El-Shemy H.A. (2014). Cytotoxic and antioxidant properties of active principals isolated from water hyacinth against four cancer cells lines. BMC Complement. Altern. Med..

[144] Roy R.N., Laskar S., Sen S.K. (2006). Dibutyl phthalate, the bioactive compound produced by Streptomyces albidoflavus 321.2. Microbiol. Res..

[145] Khatiwora E., Adsul V.B., Kulkarni M., Deshpande N.R., Kashalkar R.V. (2012). Antibacterial activity of Dibutyl Phthalate: A secondary metabolite isolated from Ipomoea carnea stem. J. Pharm. Res..

[146] Mini Shobi T., Gowdu Viswanathan M.B. (2018). Antibacterial activity of di-butyl phthalate isolated from Begonia malabarica. J. Appl. Biotechnol. Bioeng..

[147] Lee D.S. (2000). Dibutyl phthalate, an α-glucosidase inhibitor from Streptomyces melanosporofaciens. J. Biosci. Bioeng..

[148] Hoang V.L.T., Li Y., Kim S.K. (2008). Cathepsin B inhibitory activities of phthalates isolated from a marine Pseudomonas strain. Bioorg. Med. Chem. Lett..

[149] Fitzgerald D.J., Stratford M., Gasson M.J., Ueckert J., Bos A., Narbad A. (2004). Mode of antimicrobial of vanillin against Escherichia coli, Lactobacillus plantarum and Listeria innocua. J. Appl. Microbiol..

[150] Cava-Roda R.M., Taboada-Rodríguez A., Valverde-Franco M.T., Marín-Iniesta F. (2012). Antimicrobial Activity of Vanillin and Mixtures with Cinnamon and Clove Essential Oils in Controlling Listeria monocytogenes and Escherichia coli O157:H7 in Milk. Food Bioprocess Technol..

[151] Tai A., Sawano T., Yazama F., Ito H. (2011). Evaluation of antioxidant activity of vanillin by using multiple antioxidant assays. Biochim. Biophys. Acta Gen. Subj..

[152] Shoeb A., Chowta M.N., Pallempati G., Rai A., Singh A. (2013). Evaluation of antidepressant activity of vanillin in mice. Indian J. Pharmacol..

[153] Kang S.Y., Sung S.H., Park J.H., Kim Y.C. (1998). Hepatoprotective activity of scopoletin, a constituent of Solanum lyratum. Arch. Pharm. Res..

[154] Liu X.-L., Zhang L., Fu X.-L., Chen K., Qian B.-C. (2001). Effect of scopoletic on PC3 cell proliferation and apoptosis. Acta Pharmacol. Sin..

[155] Shaw C.Y., Chen C.H., Hsu C.C., Chen C.C., Tsai Y.C. (2003). Antioxidant properties of scopoletin isolated from Sinomonium acutum. Phyther. Res..

[156] Rollinger J.M., Hornick A., Langer T., Stuppner H., Prast H. (2004). Acetylcholinesterase inhibitory activity of scopolin and scopoletin discovered by virtual screening of natural products. J. Med. Chem..

[157] Ding Z., Dai Y., Wang Z. (2005). Hypouricemic action of scopoletin arising from xanthine oxidase inhibition and uricosuric activity. Planta Med..

[158] Carpinella M.C., Ferrayoli C.G., Palacios S.M. (2005). Antifungal synergistic effect of scopoletin, a hydroxycoumarin isolated from Melia azedarach L. fruits. J. Agric. Food Chem..

[159] Manuele M.G., Ferraro G., Barreiro Arcos M.L., López P., Cremaschi G., Anesini C. (2006). Comparative immunomodulatory effect of scopoletin on tumoral and normal lymphocytes. Life Sci..

[160] Panda S., Kar A. (2006). Evaluation of the Antithyroid, Antioxidative and Antihyperglycemic Activity of Scopoletin from Aegle marmelos leaves in Hyperthyroid Rats. Phyther. Res..

[161] Darmawan A., Kosela S., Kardono L.B.S., Syah Y.M. (2012). Scopoletin, a coumarin derivative compound isolated from Macaranga gigantifolia Merr. J. Appl. Pharm. Sci..

[162] Verma A., Dewangan P., Kesharwani D., Kela S.P. (2013). Hypoglycemic and hypolipidemic activity of scopoletin (coumarin derivative) in streptozotocin induced diabetic rats. Int. J. Pharm. Sci. Rev. Res..

[163] Nam H., Kim M.M. (2015). Scopoletin has a potential activity for anti-aging via autophagy in human lung fibroblasts. Phytomedicine.

[164] Nakamura Y., Murakami A., Koshimizu K., Ohigashi H. (1996). Identification of Pheophorbide a and Its Related Compounds as Possible Anti-tumor Promoters in the Leaves of Neptunia oleracea. Biosci. Biotechnol. Biochem..

[165] Cheng H.H., Wang H.K., Ito J., Bastow K.F., Tachibana Y., Nakanishi Y., Xu Z., Luo T.Y., Lee K.H. (2001). Cytotoxic pheophorbide-related compounds from Clerodendrum calamitosum and C. cyrtophyllum. J. Nat. Prod..

[166] Chan J.Y.W., Tang P.M.K., Hon P.M., Au S.W.N., Tsui S.K.W., Waye M.M.Y., Kong S.K., Mak T.C.W., Fung K.P. (2006). Pheophorbide a, a major antitumor component purified from Scutellaria barbata, induces apoptosis in human hepatocellular carcinoma cells. Planta Med..

[167] Tang P.M.K., Chan J.Y.W., Au S.W.N., Kong S.K., Tsui S.K.W., Waye M.M.Y., Mak T.C.W., Fong W.P., Fung K.P. (2006). Pheophorbide a, an active compound isolated from Scutellaria barbata, possesses photodynamic activities by inducing apoptosis in human hepatocellular carcinoma. Cancer Biol. Ther..

[168] Busch T.M., Cengel K.A., Finlay J.C. (2009). Pheophorbide a as a photosensitizer in photodynamic therapy: In vivo considerations. Cancer Biol. Ther..

[169] Bui-Xuan N.H., Tang P.M.K., Wong C.K., Fung K.P. (2010). Photo-activated pheophorbide-a, an active component of Scutellaria barbata, enhances apoptosis via the suppression of ERK-mediated autophagy in the estrogen receptor-negative human breast adenocarcinoma cells MDA-MB-231. J. Ethnopharmacol..

[170] Islam M.N., Ishita I.J., Jin S.E., Choi R.J., Lee C.M., Kim Y.S., Jung H.A., Choi J.S. (2013). Anti-inflammatory activity of edible brown alga Saccharina japonica and its constituents pheophorbide a and pheophytin a in LPS-stimulated RAW 264.7 macrophage cells. Food Chem. Toxicol..

[171] Jansen O., Tchinda A.T., Loua J., Esters V., Cieckiewicz E., Ledoux A., Toukam P.D., Angenot L., Tits M., Balde A.M. (2017). Antiplasmodial activity of Mezoneuron benthamianum leaves and identification of its active constituents. J. Ethnopharmacol..

[172] Choi D., Lim G.S., Piao Y.L., Choi O.Y., Cho K.A., Park C.B., Chang Y.C., Song Y., Lee M.K., Cho H. (2014). Characterization, stability, and antioxidant activity of Salicornia herbaciea seed oil. Korean J. Chem. Eng..

